# BitPhylogeny: a probabilistic framework for reconstructing intra-tumor phylogenies

**DOI:** 10.1186/s13059-015-0592-6

**Published:** 2015-02-13

**Authors:** Ke Yuan, Thomas Sakoparnig, Florian Markowetz, Niko Beerenwinkel

**Affiliations:** University of Cambridge, Cancer Research UK Cambridge Institute, Cambridge, UK; Department of Biosystems Science and Engineering, ETH Zurich, Basel Switzerland; SIB Swiss Institute of Bioinformatics, Basel, Switzerland; Current address: Biozentrum, University of Basel, Basel, Switzerland

## Abstract

Cancer has long been understood as a somatic evolutionary process, but many details of tumor progression remain elusive. Here, we present BitPhylogeny, a probabilistic framework to reconstruct intra-tumor evolutionary pathways. Using a full Bayesian approach, we jointly estimate the number and composition of clones in the sample as well as the most likely tree connecting them. We validate our approach in the controlled setting of a simulation study and compare it against several competing methods. In two case studies, we demonstrate how BitPhylogeny reconstructs tumor phylogenies from methylation patterns in colon cancer and from single-cell exomes in myeloproliferative neoplasm.

**Electronic supplementary material:**

The online version of this article (doi:10.1186/s13059-015-0592-6) contains supplementary material, which is available to authorized users.

## Background

Cancer is a somatic evolutionary process. Tumors are complex mixtures of heterogeneous subclones, and the genetic and epigenetic diversity within tumors can be a major cause of drug resistance, treatment failure, and tumor relapse [1,2]. Profiles of somatic mutations or DNA methylation can reveal the structure of the tumor cell population and contain traces of its past proliferative history [3-8]. Tumor subclones often display a cellular differentiation hierarchy inherited from their tissue of origin, and epigenetic changes are particularly informative about these relationships [9]. While tumor heterogeneity has been observed widely [10], an in-depth understanding of the underlying evolutionary and (perturbed) differentiation processes is lagging behind since phylogenetic trees describing the population structure of tumors are typically constructed manually [6].

Rigorous and accurate phylogenetic methods to infer automatically tumor ‘life histories’ and differentiation hierarchies from molecular profiles could have a profound impact on cancer research. For example, such methods would make it possible to infer early driver events on a large scale, to test whether evolutionary trajectories are predictive of clinical outcomes, and to compare the mode and speed of evolution between primary and metastatic tumors. Many clinical studies are currently measuring cancer heterogeneity, and robust intra-tumor phylogenetic methods are essential to interpret these data and to allow for reliable conclusions.

### The intra-tumor phylogeny problem

**Figure 1:**
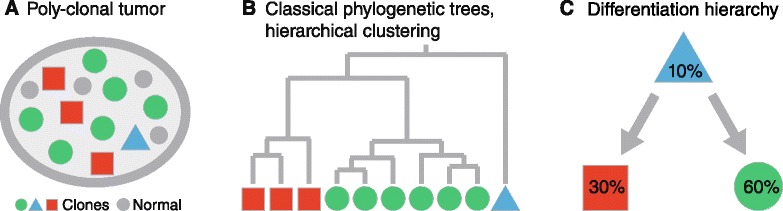
**The intra-tumor phylogeny problem.** **(A)** Molecular profiles obtained from a bulk sequenced heterogeneous tumor are shown. They consist, in this example, of three clones (red squares, blue triangles, and green discs) and normal cells (small grey discs). The intra-tumor phylogeny problem is to infer the population structure of the tumor, i.e., to identify the different clones and to elucidate how they relate to each other. **(B)** Classical phylogenetic trees and hierarchical clustering methods place the observed molecular profiles at the leaf nodes of a tree, while the inner nodes represent unobserved common ancestors. Here, leaf nodes are defined as the nodes without any child nodes and inner nodes as the nodes that have at least one child node. **(C)** Unlike classical phylogenetic tree models, BitPhylogeny clusters molecular profiles to identify subclones and places them as both inner (blue triangle) and leaf nodes (red square, green disc) of a tree describing the hierarchy of the tumor cell population.

Single-cell studies offer the most direct evidence of tumor heterogeneity, but are often limited to either a small number of genetic markers [11] and genes [12] or a small number of sequenced cells [13] with generally high error rates and high allelic dropout rates [14,15]. Thus, today, the main data source for evolutionary inference is bulk sequencing of mixed tumor samples [3,5,6], which is also the most readily available type of data for clinical applications of evolutionary methods in translational medicine. Whether obtained from single-cell or bulk sequencing, we assume in the following that the sequencing reads provide a statistical sample of the genomes of the underlying cell population. The intra-tumor phylogeny problem is to reconstruct the population structure of a tumor from these data. The problem consists of two tasks, namely (i) identifying the tumor subclones and (ii) estimating their evolutionary relationships (Figure 1).

Here, we present a unified approach to the intra-tumor phylogeny problem, called BitPhylogeny, which addresses both subproblems simultaneously. Instead of sequentially clustering and tree building, we combine both steps into a single model. Our unified model jointly solves both parts of the intra-tumor phylogeny problem and automatically (i) estimates the number of clones and (ii) places them at the leaves and inner nodes of a phylogenetic tree that reflects their evolutionary relationships. Our approach is based on non-parametric Bayesian mixture modeling using a tree-structured stick-breaking process (TSSB) [16], similar to a previous model for somatic mutations [17].

Our framework is very flexible and can be adapted to the specific requirements of the data, as we demonstrate in two case studies, one using methylation patterns and the other whole-exome single-nucleotide variant (SNV) patterns as markers of evolution. In the first case study, we focus on patterns of CpG methylation, referred to here as *methyltypes*, observed in read data obtained from bisulfite sequencing of a mixed tumor sample. DNA methylation is a somatic change that accumulates in a clock-like fashion during aging [18]. It is a particularly precise marker of cell fate because the error rate is several orders of magnitude higher than for nucleotide substitutions [19]. In neutral genomic regions, the number of somatic errors increases linearly with the number of cell divisions [9]. Neutral methylation patterns thus act as a molecular clock [18]. In the context of cancer, such molecular clocks have been used to study intra-tumor evolution in a wide range of cancers, including lymphomas [20], brain cancer [21], prostate cancer [22] and colon cancer [23-25]. Here, we use data from Sottoriva et al. [9], who collected methylation profiles of 40 glands from five colorectal tumors. For each tumor, two regions were sectioned from opposite sides of the tumor, and in each region, three to five glands were microdissected. The authors analyzed these data by a combination of spatial agent modeling and statistical analysis. They presented tumor-specific lineage trees, in which the leaf nodes (i.e., the nodes without children) are the individual methylation patterns, but they did not identify clones. Our method differs from this approach in two important aspects: BitPhylogeny identifies clonal subpopulations from a mixture model and it organizes them into leaf and inner nodes of an evolutionary tree, which directly represents the differentiation hierarchy of the tissue (Figure 1).

In the second case study, we applied BitPhylogeny to SNVs called from whole-exome sequencing of single cells. The mutational patterns from single cells, referred to as genotypes, are used to identify clones. Therefore, we directly define a clone as a set of cells sharing the same genotype. This is a key difference to using allele frequencies of SNVs from bulk-sequenced tumor samples as we discuss below. We use data from Hou et al. [14], which includes SNV genotypes of 58 single cancer cells derived from one tumor of a patient with myeloproliferative neoplasm. In this case, the phylogeny was built based on the accumulation of mutations in the entire exome, demonstrating the scalability of BitPhylogeny to large genomic regions.

### Related work

The intra-tumor phylogeny problem involves subclone identification and phylogeny reconstruction from noisy observations. For short-read DNA sequencing data obtained from mixed samples, inferring subclonal structure alone has been addressed by first calling SNVs and then grouping the SNVs into clones according to their estimated frequencies using mixture models. PyClone [26] and THetA [27] both follow this strategy and also correct SNV frequencies for copy number variations. The outcome of these analyses is the number of clones and their frequencies, but the evolutionary history of the clones remains unknown, such that some studies relied on visual inspection and manual placement of the clones in a phylogenetic tree [6].

A common approach to phylogenetic inference in human genetics and also in cancer genomics is to use a perfect phylogeny for the evolutionary relationships among haplotypes, which assumes infinite sites and no recombination. Efficient algorithms exist for computing (approximate) perfect phylogenies [28-31]. In principle, these methods can also be used for reconstructing intra-tumor phylogenies. However, perfect phylogeny algorithms typically lack a probabilistic interpretation and they assume a given and fixed number of haplotypes. For tumor phylogenies, these assumptions need to be relaxed. In a recent attempt to achieve this goal, SNV data were used iteratively to construct perfect phylogenies, identify subclones and improve SNV calling [32]. Other recent applications of classical phylogenetic inference methods [33] place mutation patterns [14,15], copy-number aberrations [34] or methylation profiles [9] at the leaves of a tree, without clustering them into clones or placing them at inner nodes (i.e., the nodes with children).

Because tumor subclones may also occur at inner nodes of phylogenetic trees [2,6], the applicability of classical phylogenetic methods is limited. A recent approach, called TrAp [35], addresses subclonal deconvolution and phylogenetic inference jointly by solving a highly constrained matrix inversion problem. It takes as input the population frequencies of a limited number of aberrations (up to 25) and deconvolves them in a linear combination of subclones that are connected in a tree. In principle, TrAP could be used as a follow-up step to clustering SNVs with an approach like PyClone. However, the clustering and tree-building steps are not independent and decoupling them may result in suboptimal performance if initially established clusters need to be spread out over different parts of the tree [6]. Additionally, TrAP cannot be easily applied to methylation data, as we use here, because it does not take back-mutations into account.

A first unified approach to combine clustering and tree inference with clones at inner nodes is PhyloSub [17], which is based on non-parametric Bayesian mixture modeling using a TSSB [16]. PhyloSub uses SNV frequency data to inform the tree topology and is thus subject to the limitations that this type of data exhibits. For example, clusters with few observations, i.e., small subclones, are difficult to identify and their placement in the tree topology is highly uncertain if only frequency constraints are used for tree construction. For example, in Nik-Zainal et al. [6], one of the clusters was spread over three branches of the tree after manual construction of the tree and refinement by incorporating information from the few reads which covered more than one SNV.

The inherent limitation of using SNV data for phylogenetic inference is that the phasing of mutations relies only on their frequencies. SNV frequencies are difficult to estimate from noisy sequencing data, especially if the coverage is low, and in general different clones may have identical or very similar frequencies. The phasing of SNVs can be improved with longer reads, such that multiple co-occurring SNVs are observed on the same read. For example, for RNA viruses, which display much more genetic diversity than tumors, overlapping reads have been used successfully to reconstruct long-range haplotypes from mixed samples [36]. With constantly improving sequencing technologies, including increasing read length and single-cell approaches, we expect that more data with these characteristics will be widely available in the near future.

For methylation patterns in cancer, computational modeling dates back at least to Siegmund et al. [37], who assumed that unique methylation patterns are generated from a hidden phylogenetic tree. Such trees were reconstructed from data using approximate Bayesian computation. A recently proposed strategy [38] uses a given phylogenetic tree structure to estimate the true methylation patterns under the assumption that observed methylation patterns are noisy and sometimes completely missing.

In addition to SNV and methylation data, gene expression profiles have been used to reconstruct phylogenies of tumor samples [39,40]. In particular, Desper et al. [39] regard tumor samples as representing different stages in the progression of the disease, and reconstruct the history of these stages using phylogenetic approaches. Riester et al. [40] built phylogenies based on gene expression profiles from cancer subtypes. Along this line of research, attempts have been made to cluster samples together and to build phylogenies based on the clusters. Hierarchical clustering [41], the Dirichlet process [42] and matrix factorization [43] have been proposed for clustering. To arrange clusters into a tree, all of these methods constructed minimum spanning trees.

At the single-cell level, Pennington et al. [44] employed fluorescent *in situ* hybridization (FISH) data for phylogenetic inference. Their tree-building method allows internal (so-called Steiner) nodes. Finally, copy number data from single cells were employed to construct phylogenies using the neighbor-joining method [45]. Both approaches built phylogenetic trees for single cells, whereas here, we focus on building such trees for clones, i.e., for collections of cells.

## Results and discussion

**Figure 2:**
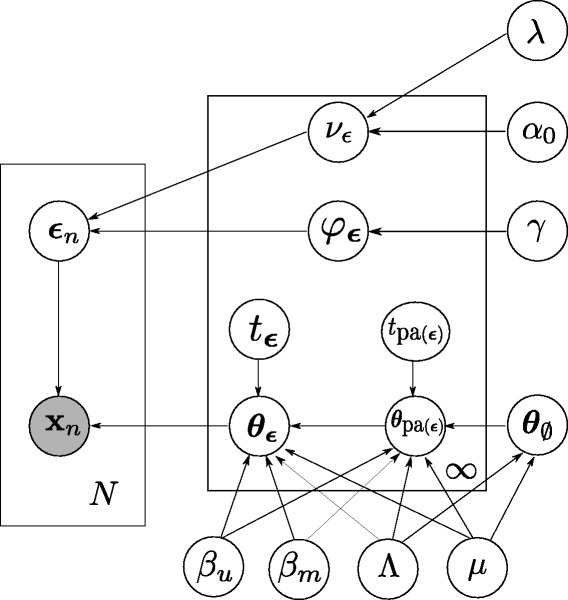
**BitPhylogeny** **as a graphical model.** Each of a total of *N* observed marker patterns is denoted by x_*n*_ (shaded node). The clone membership of each observation is denoted by *ε*_*n*_ and generated by a tree-structured stick-breaking process with variables *ν*_*ε*_ (clone size) and *φ*_*ε*_ (branching probability), and parameters *λ*, *α*_0_ and *γ*. For each clone, *t*_*ε*_ and *θ*_*ε*_ are the branch length and clone parameter, respectively, which determine the local probability distribution of observing a marker pattern from this clone. The function pa(·) denotes the parent of each clone in the tree, except the root clone *∅*. The transition probabilities *p*(*θ*_*ε*_∣*θ*_pa(*ε*)_) have hyperparameters *β*_*m*_, *β*_*u*_, *Λ* and *μ*.

We have developed BitPhylogeny (Bayesian inference of intra-tumor phylogenies), an integrated approach to address the intra-tumor phylogeny problem. The statistical model is based on simultaneously assigning markers of evolution to clones, which are represented as both inner nodes and leaves of a phylogenetic tree, and on learning the topology and the parameters of the tree. We use a TSSB to construct a prior probability of trees and a Markov chain Monte Carlo (MCMC) inference scheme for sampling from the joint posterior. The relationships between parent and child nodes are derived from a classical phylogeny model. The model is formally defined in Materials and methods and depicted as a graphical model in Figure 2. In the following, we benchmark BitPhylogeny in simulation studies and discuss its application to colon cancer methylation data.

### Validation in simulation studies

**Figure 3:**
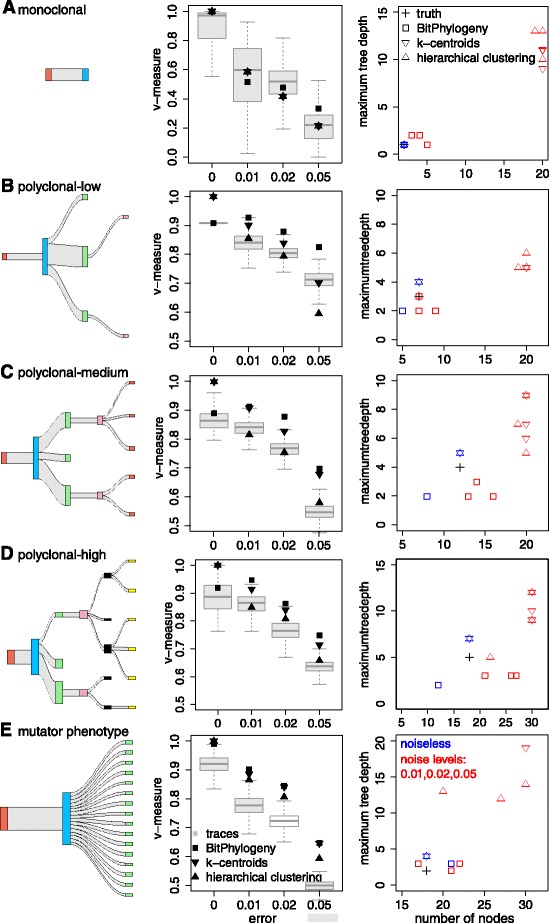
**Simulation study with five trees (A-E).** **(First column)** Sankey plots of the trees used for simulations. For each node, the width of the in-edge is proportional to the clone frequency. The colors denote different layers of the tree (tree depths). Plots were produced with the R package riverplot. **(Second column)** Performance of clustering methods for the simulation studies with four different noise levels. Performance measures are based on 10,000 MCMC samples (the box plots in the second column). The MPEAR-summarized predictions (marked as BitPhylogeny) outperform the baseline competitors in all data sets with noise. **(Third column)** Comparison in terms of the summary statistics maximum tree depth and number of clones. For hierarchical clustering and k-centroids, the trees are constructed as minimum spanning trees from estimated clonal methyltypes.

We have assessed the performance of BitPhylogeny in the controlled settings of five simulation studies. Based on the review article by Navin and Hicks [46], we chose the simulated trees to be representative of different modes of evolution (Figure 3). One tree reflects monoclonal evolution, three trees are based on a polyclonal mode of evolution, and one tree assumes a mutator phenotype.

For monoclonal evolution, we constructed a tree that has only two clones. The root node represents healthy cells and the child clone of the root represents a monoclonal tumor (Figure 3A). A monoclonal hierarchy has been predicted to be the dominant mode of evolution in a *JAK2*-negative myeloproliferative neoplasm single-cell sequencing study [14]. For the polyclonal trees, we first constructed a tree similar to the manually reconstructed tree presented in [5] (polyclonal-low, Figure 3B). This tree was originally based on whole-genome sequencing data with an average coverage of only 188 and hence limited power to detect small clones [5]. Since many more small clones are expected in tumors [47], we added small clones to the tree. Specifically, we added another tree level with five clones; three had relative frequency 0.03 and the other two had 0.02 (polyclonal-medium, Figure 3C). This tree was further extended to contain 18 clones, most of them having a very low frequency (around 0.02) (polyclonal-high, Figure 3D). Finally, the mutator phenotype mode of evolution is represented by a star-like tree with many leaf nodes (Figure 3E). Evidence for this model, which is driven by high mutation rates, has been found in multiple neoplastic tissues [48].

For each of the five trees, we simulated eight marker sites, sampled 2,000 observations, and added observation errors by flipping every site with a fixed error probability of 0, 0.01, 0.02 or 0.05 in four separate simulation runs. We subsequently applied BitPhylogeny and evaluated its performance for each MCMC iteration separately. The first 30,000 iterations were discarded as a burn-in phase, and the next 50,000 samples were collected and thinned out by a factor of 5. The following analyses are based on the resulting 10,000 samples.

#### Clustering performance

The clustering performance of BitPhylogeny was compared to that of two baseline methods: k-centroids and hierarchical clustering with model selection using silhouette scores (see Materials and methods). We used the v-measure to compare the clustering performance of the three methods (see Materials and methods). The BitPhylogeny MCMC samples (called the trace) are summarized by the MPEAR approach, which generates an optimal clustering configuration from the samples (see Materials and methods). For perfect data without errors, all three methods achieved perfect performance for the monoclonal tree. BitPhylogeny achieved almost perfect clustering for the mutator tree (Figure 3A,E, second column). However, for the three polyclonal trees (low, medium and high), the two baseline methods achieve perfect clustering, while BitPhylogeny comes very close to perfect performance with a v-measure of around 0.9 (Figure 3B,C,D, second column). This is because some of the clones have very similar marker profiles (e.g., a single differing marker site), and highly asymmetric sizes. As a result, in these cases, BitPhylogeny tends to underestimate the number of clusters, since a mixed clone is more preferred than separated clones. However, importantly, the advantage of using BitPhylogeny is very clear in the presence of noise. The MCMC traces from BitPhylogeny perform similar to k-centroids and hierarchical clustering and the MPEAR-summarized results outperform the baseline methods at all noise levels.

#### Tree reconstruction performance

We compared BitPhylogeny to minimum spanning trees constructed from the clustering results obtained by the two baseline clustering methods. The third column of Figure 3 shows tree depth and number of nodes as summary statistics for all trees at all noise levels in all simulation studies. Overall, the trees reconstructed from the baseline clustering methods have too many clusters and are much deeper than the true tree (Additional file 1: Figure S2).

**Figure 4:**
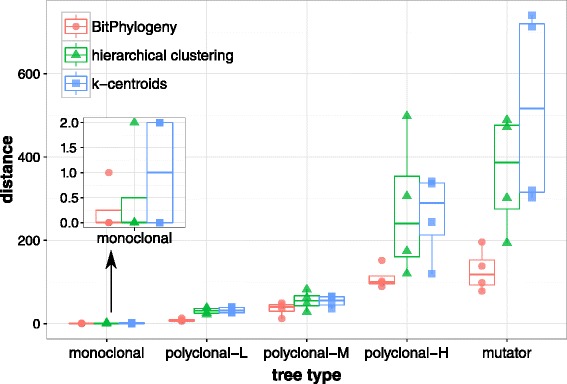
**Consensus node-based shortest path distances for all simulated trees.** Each box plot is summarized for the distance measures across four noise levels (0*%*, 1*%*, 2*%* and 5*%*). The suffixes L, M and H for the polyclonal tree type refer to the polyclonal-low, -medium and -high trees in Figure 3. BitPhylogeny consistently outperforms the two baseline methods.

To compare the tree topologies explicitly, we developed a distance measure called *consensus node-based shortest path distance* (see Materials and methods for details). The performance of BitPhylogeny is examined based on the empirical MAP solution (see Materials and methods). The results for all synthetic data sets (five clone compositions and four noise levels) are presented in Figure 4. For all clonal compositions and noise levels, BitPhylogeny constructs trees that are much closer to the true tree than both baseline methods. For the monoclonal tree, all three methods are able to reconstruct the two clones accurately. However, as clonal composition becomes more complex, the performance of the two baseline methods starts to degrade quickly. The baseline methods overestimated the number of clones and produce much deeper trees for most synthetic data sets. As a result, they perform poorly when the complexity of clone composition increases.

### Case study 1: colon cancer development

We applied BitPhylogeny to bisulfite sequencing data using the 454/Roche technology of the *IRX2* locus of colon tumors from [9]. The tumors from different patients are denoted as CT, CU, CX and HA. Between three and five samples are available from two spatially separated sides of the tumor (denoted left and right). Each sample is denoted by the tumor, the tumor side and a number. For example, CT_R1 is the first sample from the right side of tumor CT. On average, there are more than 1,500 reads per cancer gland. The methylation tag sequencing fidelity was 99.6%, i.e., the data exhibit an error rate of 0.4% [9].

To compare trees from different samples and tumors, we analyzed a number of topological features of the BitPhylogeny trees. The number of big clones (carrying more than 1% of the tumor mass) and the total branch length are measures of the heterogeneity of the samples. The maximum depth of the tree and the root-to-leaf mass distribution are indicators of the level of differentiation of the tumor sample. The trees of most samples have between two and four node levels, or tree layers. In general, we find that the number of big clones correlates with the maximum depth of the trees. The root clones of the trees contain up to 30% of the tumor mass with a strong bias for small root nodes (median 2.3% of the tumor mass). A notable exception is CX_R6 with 36%. With a median of 62% across all samples, most of the mass is usually assigned to the second layer of the trees. However, this value ranges from 7% to 98%. The third layer has a median mass of 25% and the fourth layer 2%.

#### Analysis of CT and the CX samples

**Figure 5:**
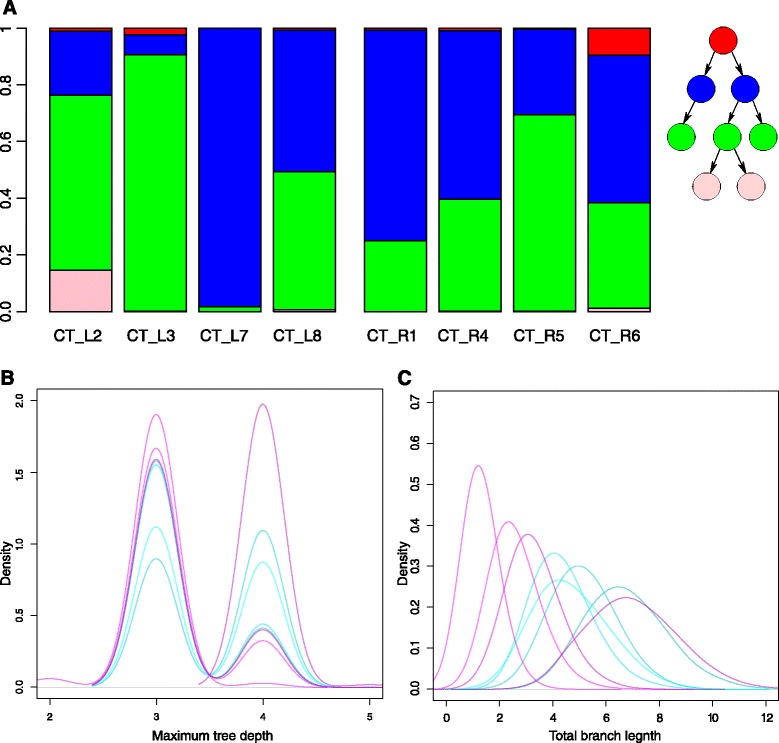
**Analysis of CT samples.** **(A)** Level-wise mass distribution of CT samples. For each tree, the bars show the level sums of the mixture model masses for all eight samples of the CT tumor. The red bars correspond to the posterior means of the root masses. The blue, green and pink bars correspond to the means of the sums of the second, third and fourth tree levels, respectively. **(B)** Maximum depth of trees of the individual samples. Turquoise densities are from the right side of the tumor and the pink ones are from the left side. Trees from the left side of the tumor have peaked posterior densities at a depth of either 3 or 4, while the posterior densities from the right side are less peaked. **(C)** Total branch length of trees of individual samples. The trees from the left side, which peak at depth 3 in (B), have shorter total branch lengths than the tree that peaks at depth 4 or the trees from the right side of the tumor.

Among the CT samples, we find that the left side displays more topological diversity than the right side (Figure 5). The samples from the right side have most of their mass assigned to levels two and three, though the tumor mass in the left side in one sample, CT_L7, is mostly at the second level. In CT_L3, most of the tumor mass is at the third level and in CT_L2 a considerable proportion of the tumor mass, about 18%, is assigned to the fourth level. The fourth sample of the left side, CT_L8, exhibits a mass distribution similar to the right side. The pairwise symmetrized Kullback–Leibler divergences, a measure of topological diversity, among samples from the left colon side were larger than those within the right side (*P*=0.002, Wilcoxon rank sum test).

In terms of maximum tree depth, two samples from the right side show a uniform behavior between 3 and 4 and the remaining two have a bias towards a maximum depth of 3. The samples from the left side, however, all have a strong tendency for a maximum depth of either 3 (three samples) or 4 (one sample: CT_L2). The variance of the posterior of the maximum depth is bigger for the right side than for the left side (*P*=0.014, Wilcoxon rank sum test). The same behavior can be observed for total branch length. The same three samples that show a bias towards three node levels also have a shorter total branch length than the samples from the right side (Figure 5). These summary statistics indicate that the right side of the tumor evolved at a more homogeneous speed than the left side.

**Figure 6:**
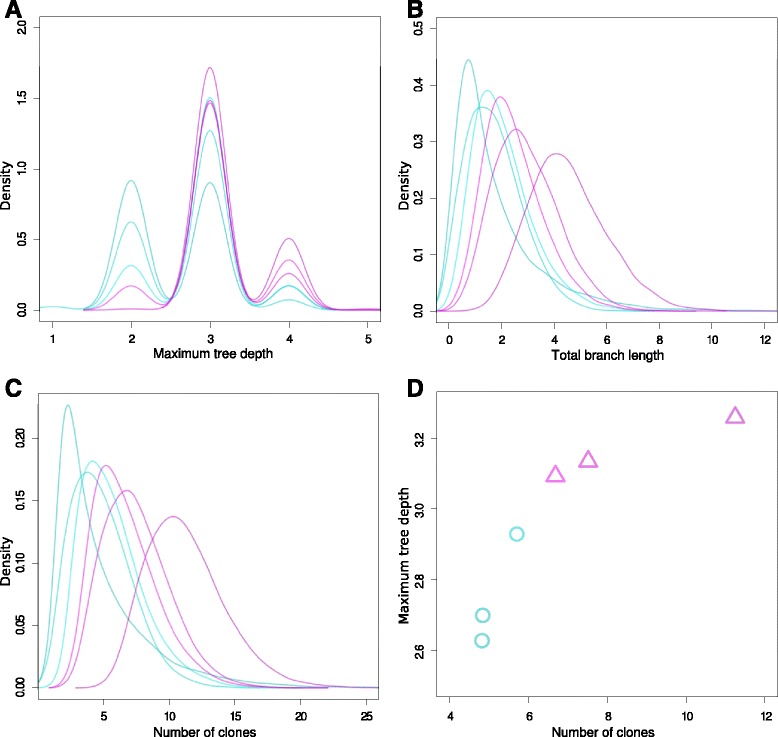
**Analysis of CX samples and joint analysis of all samples.** Turquoise densities are for samples from the right side of the tumor and the pink ones are for the left side. **(A)** Maximum depth of trees. Trees from the right side have posterior maximum depth between 2 and 3, while trees from the left side have posterior maximum depth between 3 and 4. **(B)** Total branch length of trees. Trees from the right side have slightly shorter total branch lengths than the trees from the left side. **(C)** Number of clones in a tree. Trees from the right side contain fewer clones than trees from the left side. **(D)** Mean number of clones versus mean maximum depth of trees. With these two summary statistics of trees, samples from the left and right can be separated very clearly.

For the CX sample, we observed that the left side can be well separated from the right side in terms of maximum tree depth (*P*=0.027, *t*-test; Figure 6). Less pronounced, this separation was also found for the total branch length as well as for the number of clones and big clones. In the original study [9], the CX sample was identified as the largest tumor in the study. The size of this tumor could be a reason for the separation of the evolutionary history of the right side from the left side. Additionally, they identified CX as the tumor with the highest cancer stem cell fraction. We did not observe such a clear separation of left and right sides in any of the other samples. The left side of the tumor appears to evolve faster than the right side, because it has deeper trees with more clones and longer total branch lengths than the right side.

The phylogenetic trees in Sottoriva et al. [9] do not contain an error model, i.e., every distinct methylation pattern is considered a clone, and therefore they cannot be compared to our BitPhylogeny trees. The authors did not attempt to reconstruct hierarchical relationships between clonal subpopulations, but rather estimated the fraction of cancer stem cells in the tumor and the degree of heterogeneity. They found a large degree of intra-tumor and intra-sample heterogeneity as well.

### Case study 2: myeloproliferative neoplasm

We used BitPhylogeny to analyze single-cell sequencing data of a *JAK2*-negative myeloproliferative neoplasm [14]. The data set consists of 712 SNVs detected by sequencing the exomes of 58 cancer cells. In addition to single cells, the data set also contains genotypes from bulk-sequenced normal and tumor tissue. On average, Hou et al. [14] estimated the allele dropout rate to be 43.09*%* and the false discovery rate to be 6.04×10^−5^. Thus, a large amount of data is missing (on average 56.13*%* per cell). We binarized the SNV profile by writing 0 for the wild-type allele and 1 for a heterozygous mutation.

**Figure 7:**
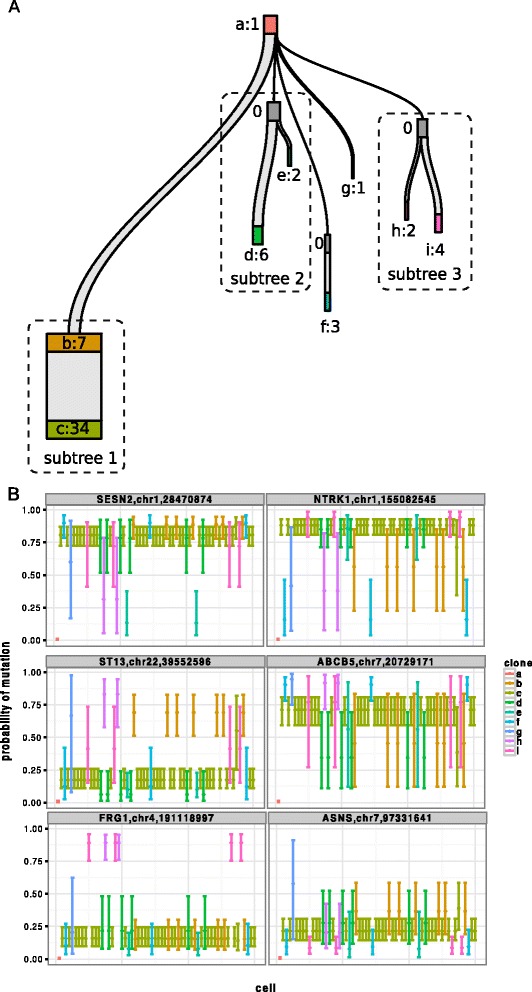
**Reconstructed tree and mutation profiles from single-cell exome sequencing data.** **(A)** Reconstructed phylogeny. Non-empty clones are labeled *a* through *i* followed by the number of cells they contain. The vertical distance represents the evolutionary distance between clones. **(B)** Estimated probabilities of six SNVs in key genes across all cells. The error bars summarize 50,000 MCMC samples and are color-coded according to clone membership.

Figure 7A presents the results of the BitPhylogeny analysis. It shows a tree structure with a major clone (labeled as clone *c*) containing 33 out of all 58 cells. This clone is the most progressed clone since it has the longest total branch length from the root clone. One distinct feature of the reconstructed tree is that it captures both clonal progression (e.g., clone *b* to *c*) and binary branching with unobserved common ancestors (e.g., clones *d* and *e*). As a validation, genotypes from both bulk-sequenced normal and cancer cells are included in the analysis. The normal genotype is correctly identified as the root of the tree (clone *a* in Figure 7A). The genotype of the bulk-sequenced tumor is assigned to the most progressed clone *c*. While the analysis of the data with classical phylogenetic models in the original study only showed evidence for monoclonal evolution, BitPhylogeny reveals an additional structure of the tumor phylogeny involving in total half of all cells analyzed.

In addition to the phylogeny, BitPhylogeny provides genotype estimates for all loci and cells. This is useful due to the high amount of missing data and the high error rate of the sequencing data. Hou et al. [14] identified eight key genes that are mutated within the tumor of the patient. With BitPhylogeny, we could gain more insight into the role of these mutations in the progression of the disease. Figure 7B shows the genotype estimations of six SNVs that are located in these key genes. The full profiles of genotype estimations for all eight genes are provided in Additional file 1: Figure S3. *SESN2*, which is related to DNA damage and genetic instability, was the top gene of interest in the analysis of Hou et al. [14]. Our results confirm this finding as the gene is mutated in most cells. Interestingly, mutations in *NTRK1* and *ABCB5* may play a role during the expansion from clone *b* to *c*. The lower bounds of the error bars for clone *b* of these two genes are below 0.25, indicating only a 25*%* chance of being mutated. In contrast, clone *c* clearly has these two SNVs. The genotype profiles also suggest that the SNV in *FRG1* (which may be involved in pre-mRNA splicing [14]) are private to the clones in subtree 3. Unlike Hou et al., our results suggest that *ASNS* is not mutated, because the estimated probability of mutation for the corresponding SNV is very low for most cells.

The SNV in gene *ST13* (Figure 7B) is mutated in clone *b*, but not in clone *c*. Since clone *b* precedes clone *c*, this violates the infinite sites assumption for point mutations (which does not allow back mutations). An explanation for this phenomenon could be the following: BitPhylogeny accounts for two different sources of uncertainty, namely the stochasticity of the evolutionary process itself and observational noise. At the evolutionary level, BitPhylogeny does not allow mutations to revert back to normal. At the observational level, however, base changes can be due to sequencing errors or missing entirely. Therefore, a violation of the infinite sites model can occur in the tree if the observational likelihood outweighs the evolutionary model. In such cases, the error bars may indicate how the data are explained by the model. In the present case, the error bars for the genotype of clone *b* are relatively large. The lower bound reaches 0.5, indicating only a 50*%* chance of being mutated. In addition, it could also be the case that the mutation is present in the cells of clone *c*, but are miscalled because of insufficient coverage at the site.

### Modes of tumor progression

The topology of intra-tumor phylogenetic tress can provide insights into the mode of evolution of the tumor [46]. Like classical phylogenetic trees, BitPhylogeny trees are expected to show distinct structural features for different modes of tumor progression.

For monoclonal tumors (Figure 3A), the BitPhylogeny tree would be expected to consist of a single homogeneous clone. In both methylation and single-cell data, we did not find trees supporting this mode of tumor progression in any of the samples we analyzed. However, we observed patterns reminiscent of monoclonal evolution at the subtree level. For example, in subtree 1 of the single-cell tree (Figure 7), progression from clone *b* to *c* can be regarded as a monoclonal evolutionary pattern. Clone *c* arises from clone *b* through the acquisition of additional SNVs, some of which may provide a selective advantage. The linear subtree structure without branching suggests that clone *c* replaces clone *b* in a clonal expansion. In contrast, a polyclonal mode of tumor progression (Figure 3B,C,D) would result in BitPhylogeny trees with a moderate number of clones distributed over a small number of tree levels, and most of the samples we analyzed fell into this category.

A mutator phenotype (Figure 3E) would generate an extreme case of polyclonality with a very large number of clones, each carrying a very small proportion of the tumor mass. We did not find any trees based on methylation data that would be consistent with this mode of tumor progression. For the single-cell SNV tree (Figure 7), there are several small clones (*d* to *i*) with similar distances to the root, which could indicate a mutator phenotype given the total number of inferred clones. However, considering that measurements have been exome-wide, the number of small clones does not appear to imply a mutator phenotype for this tumor. Confirmation of this mode of tumor progression would require data from a considerably larger population of single cells.

The cancer stem cell model has been widely discussed and the original study of Sottoriva et al. [9] is based on this assumption. Under a stem cell model, our trees capture the developmental hierarchies of stem cells. In this case, the marker profiles mainly reveal the differences among stem cell generations. The non-stem cell descendants are expected to have similar marker profiles as their immediate stem cell ancestors, which means that the stem cell lineage is driving tumorigenesis [9]. Thus, each clone would be a mixture of stem cells and their descendants, and the phylogenetic trees represent the stem cell hierarchies.

## Conclusions

BitPhylogeny provides a probabilistic framework for inferring intra-tumor phylogenies from observed sequence data. It jointly estimates the subclonal structure of a tumor and the evolutionary relationship of its clones using a full Bayesian approach. In two case studies, we have shown that the method can be useful for reconstructing the life histories of tumors and for making inference about the mode of tumor evolution. Unlike previous methods for unphased short-read data, BitPhylogeny does not rely on mutation frequencies, but rather considers haplotype sequences, specifically patterns of co-occurring genetic or epigenetic variations. More phased data can be expected to be widely available soon due to technological advances increasing the length of reads. BitPhylogeny is also applicable to other data types, like somatic copy number aberrations, which have been used for classical phylogenetic inference [34].

The present study has also revealed limitations of our approach. The data from Sottoriva et al. [9] is unique, and while the *IRX2* locus has been specifically selected for its suitability as a molecular clock, it is important to note that the information contained in a single locus might not reflect the complete evolutionary history of a tumor. Additionally, the MCMC inference scheme we use is state-of-the-art but slow for large instances, such that improved (approximate) inference methods should also be considered. Another limitation is that we assume independence of sites, which might be violated even for well-chosen molecular clocks. This limitation is shared with most classical phylogenetics methods. It can be overcome using hidden Markov models [49] or a finite-state transducer [34,50], and we plan to extend our method in this direction in the future.

A major challenge we identified is how to compare evolutionary trees with different numbers of nodes and different node labels and compositions. In this study, we have used simple summary statistics, since a general distance measure between trees of tumor evolution is lacking. Ideally, such a measure would combine the overlap between node content, as computed, for example, by the v-measure [51], with a measure of graph similarity, for example, by adapting graph alignment approaches [52]. Advances in tree comparison would not only be important for method comparison, but also for constructing and comparing evolutionary trajectories of tumors in time. Combined with tree comparison methods, approaches like BitPhylogeny could then be applied to large collections of molecular tumor profiles to identify conserved evolutionary trajectories or developmental pathways differentiating good from poor responders, which may lead to further insights into cancer evolution and progression and eventually inform treatment decisions.

## Materials and methods

BitPhylogeny employs a non-parametric Bayesian clustering approach for reconstructing intra-tumor phylogenies from observed sequence data. To integrate the assignment of sequences to clones with the organization of clones into a tree, it uses the TSSB as a prior [16]. In this model, the observed data (i.e., sequences) are associated with all nodes of the tree, rather than only to the leaves as in classical phylogenetic models. The TSSB is a probabilistic mixture model with an infinite number of hierarchically organized components, but only a finite number of components have a non-zero weight. In addition to this prior, the full generative probabilistic model underlying BitPhylogeny is defined by node-wise data distributions, a transition kernel and the root prior.

Let *i*∈{1,…,*M*} denote the considered marker sites and *n*∈{1,…,*N*} index the observed marker profiles or sequences (methyltypes or genotypes). We denote the marker state at site *i* of sequence *n* by *x*_*i*,*n*_∈{0,1}, where *x*_*i*,*n*_=1 indicates methylation or mutation. To assign sequences to clones, each clone has a label. Following the notation in [16], the clone label is defined as ***ε***=(*ε*_1_,…,*ε*_*K*_), where $\epsilon _{k} \in \mathbb {N}$ for all *k*∈{1,…,*K*}. Each label is a sequence of natural numbers indicating the location of the clone in the phylogenetic tree. For example, the clone ***ε***=(1,2) is located in the second layer of the tree, and its lineage trace is *∅*→1→2, where the numbers are node labels in each level and the root node is labeled by the empty sequence, *∅*. The length *K*=|***ε***| is the depth of clone ***ε***; for example, |(1,2)|=2. The root clone has depth |*∅*|:=0. The probability of an observed marker pattern originating from clone ***ε*** is denoted by *π*_*ε*_. This parameter specifies the proportion of observations explained by clone ***ε*** (Figure 1C).

### Tree-structured stick-breaking process

The TSSB generates an infinite series of clone proportions *π*_*ε*_, which sum to one by interleaving two stick-breaking processes [16],

(1) $$ \begin{aligned} \nu_{\boldsymbol{\epsilon}} &\sim \text{Beta}(1,\lambda^{|\boldsymbol{\epsilon}|} \alpha_{0}), \qquad \psi_{\boldsymbol{\epsilon}} \sim \text{Beta}(1,\gamma), \\ \varphi_{\boldsymbol{\epsilon}\epsilon_{i}} &= \psi_{\boldsymbol{\epsilon}\epsilon_{i}} \prod\limits_{j=1}^{\epsilon_{i}-1}(1-\psi_{\boldsymbol{\epsilon}j}), \qquad \pi_{\emptyset} = \nu_{\emptyset}, \\ \pi_{\boldsymbol{\epsilon}} &= \nu_{\boldsymbol{\epsilon}} \varphi_{\boldsymbol{\epsilon}} \prod\limits_{\{\boldsymbol{\epsilon}^{'}\}}\varphi_{\boldsymbol{\epsilon}^{\prime}} (1 - \nu_{\boldsymbol{\epsilon}^{\prime}}). \end{aligned}  $$

The beta-distributed random variables *ν*_*ε*_ and *ψ*_*ε*_ define the clone size *π*_***ε***_ by distributing mass between each node and its descendants and its siblings, respectively. The index *εε*_*i*_ denotes the *ε*_*i*_-th child of clone *ε*, and in the second product, the index *ε*^′^ runs over all ancestors of clone *ε* in the tree. The clone size depends on the depth and a decay constant *λ*. We refer to the distribution of clone sizes ***π***={*π*_*ε*_} and their hierarchical structures generated by (1) as TSSB (*λ*, *α*_0_, *γ*). Using this tree partition prior of unbounded depth and width, the infinite mixture model for the observed methylation or mutation data $\mathbf {x}_{n} = \{x_{i,n}\}_{i=1}^{M} $ is defined as

(2) $$ \begin{aligned} &\boldsymbol{\pi} \sim \text{TSSB} (\lambda, \alpha_{0}, \gamma), \qquad \epsilon_{n} \mid \boldsymbol{\pi} \sim \text{Discrete}(\boldsymbol{\pi}),\\ &\boldsymbol{\theta}_{\epsilon} \sim p(\cdot \mid \boldsymbol{\theta}_{\text{pa}(\epsilon)}), \qquad \mathbf{x}_{n} \sim p(\cdot \mid \epsilon_{n},\, \boldsymbol{\theta}_{\epsilon}), \end{aligned}  $$

where $\boldsymbol {\theta }_{\epsilon }=\{\theta _{i,\epsilon }\}_{i=1}^{M}$ are the parameters of the local distribution *p*(**x**_*n*_∣***θ***_*ε*_) of the data emitted from clone *ε*. Each clonal parameter is sampled from a transition distribution that depends on the parent clonal parameter ***θ***_pa(*ε*)_. We denote the distribution of the root parameter by *p*(***θ***_*∅*_).

### Customized methylation model

To model methylation data, we specify the local probability distributions *p*(**x**_*n*_∣***θ***_*ε*_) and the transition probabilities *p*(***θ***_*ε*_∣***θ***_pa(*ε*)_). For each clone *ε*, the local data distribution is a Bernoulli distribution with the parameter transformed by a sigmoid function. The parameter $\theta _{i, \epsilon } \in \mathbb {R}$ is used to control the probability of observing a methylation event at locus *i*. Assuming independence among loci, we set

(3) $$ \begin{aligned} p(\mathbf{x}_{n} \mid \boldsymbol{\theta}_{\epsilon}) = \prod\limits_{i=1}^{M} \sigma(\theta_{i,\epsilon})^{x_{i,n}} (1-\sigma(\theta_{i,\epsilon}))^{1-x_{i,n}}, \end{aligned}  $$

where *σ*(*θ*_*i*,*ε*_)=1/(1+ exp(−*θ*_*i*,*ε*_)). Here, we assume the CpG sites are independent, which is appropriate for the IRX2 molecular clock (verified in Additional file 1: Figure S1).

For the transition probabilities, we use a mixture of two Laplace distributions to model the parent–child relation,

(4) $$ \begin{aligned} p\left(\boldsymbol{\theta}_{\epsilon} \mid \boldsymbol{\theta}_{\text{pa}(\epsilon)}, \mu, \Lambda, \mathbf{w}\right) &=\prod\limits^{N}_{i = 1} w_{i} \, \text{Laplace}(\mu, \Lambda) \\ &\quad+ (1-w_{i})\,\text{Laplace}(-\mu, \Lambda), \end{aligned}  $$

where *μ* defines the location of a positive and a negative mode and $\mathbf {w} = \{w_{i}\}_{i=1}^{M}$. Intuitively, the positive mode generates parameters that give a high probability of observing methylation events, whereas the negative mode has the opposite effect. The hyperparameter *Λ* models variation within the modes. The weights *w*_*i*_ and 1−*w*_*i*_ of the two Laplace densities specify the probabilities of either mode being selected for sampling the child parameter. The Laplace densities have the effect of pushing the sampled parameters close to the modes *μ* or −*μ*.

The dependency on the parent parameter is introduced through the weights *w*_*i*_ as

(5) $$ w_{i} =\left\{ \begin{array}{ll} P(\theta_{i,\epsilon} \geq \eta \mid \theta_{i,\text{pa}(\epsilon)} \geq \eta) & \text{if} \,\,\,\theta_{i,\text{pa}(\epsilon)} \geq \eta \\ P(\theta_{i,\epsilon} \geq \eta \mid \theta_{i,\text{pa}(\epsilon)} < \eta) &\text{if} \,\,\,\theta_{i,\text{pa}(\epsilon)} < \eta \end{array} \right.  $$

where *η* is a fixed threshold. We set *η*=1, which results in very conservative methylation calls.

The probabilities in Equation (5) provide a link to evolutionary models used in classical phylogeny. We define a transition probability matrix **P**_*ε*_ to describe the state transition from parent to child. Let *m* denote the methylated state defined by *θ*_*i*,*ε*_≥*η* and *u* the unmethylated state. Then the matrix **P**_*ε*_ can be written as

(6) $$ \mathbf{P}_{\epsilon} = \left(\begin{array}{ll} P_{u\rightarrow u} & P_{u\rightarrow m}\\ P_{m\rightarrow u} & P_{m\rightarrow m} \end{array} \right),  $$

and according to Equation (5), we have

(7) $$ w_{i} =\left\{ \begin{array}{ll} P_{m\rightarrow m} &\text{if} \,\,\,\theta_{i,\text{pa}(\epsilon)} \ge \eta\\ P_{u \rightarrow m} &\text{if} \,\,\,\theta_{i,\text{pa}(\epsilon)} < \eta. \end{array} \right.  $$

The transition matrix is obtained from a rate matrix **A** as the matrix exponential **P**(*t*)= exp(**A***t*). The rate matrix **A** is parameterized as

(8) $$ \mathbf{A} = \left(\begin{array}{rr} -\beta_{m} &\beta_{m}\\ \beta_{u} & -\beta_{u} \end{array} \right) \rho,  $$

where *β*_*m*_ and *β*_*u*_ are the equilibrium frequencies of the methylated and unmethylated states, respectively, and *β*_*m*_+*β*_*u*_=1. The scaling factor *ρ* is set to ensure that the average rate of methylation is one, i.e., 2*β*_*u*_*β*_*m*_*ρ*=1. For each clone *ε*≠*∅*, we denote its branch length by *t*_*ε*_. Finally, the root prior is defined as

(9) $$ p(\theta_{0}) = \prod\limits^{N}_{i = 1} \text{Laplace}(-\mu, \Lambda).  $$

This prior favors clones with unmethylated states at all loci as the root.

The complete generative probabilistic model underlying BitPhylogeny, including all parameters and all conditional independencies, is depicted in Figure 2 as a graphical model.

### Customized single-nucleotide variant model

Single-cell data sets often have high rates of missing data. This can be the result of allele dropout and low depth at some regions of the genome. In [14], the authors reported that the allele dropout rate of their sequencing technique is independent of the location or base type of the locus. This matches the statistical description of data as missing completely at random. In our likelihood-based approach, the missing data can be handled by simply ignoring the locus in each cell where it is missing.

To specify the transition model for SNV data, we employ the following rate matrix,

(10) $$ \mathbf{A} = \left(\begin{array}{rr} -\beta_{m} &\beta_{m}\\ 0 & 0 \end{array} \right) \rho,  $$

where *β*_*m*_ is the frequency of a locus being mutated. The scaling factor *ρ* is set to ensure *β*_*m*_(1−*β*_*m*_)*ρ*=1. After matrix exponentiation, the probability of transition from mutation to normal is 0, which reflects the common assumption of mutations being irreversible during tumor evolution.

### Inference

For statistical inference, we pursue a Bayesian approach and estimate the full posterior probability distribution of all model parameters, including clone assignment and tree structure. For approximating the joint posterior

(11) $$   p\! \left(\{ \epsilon_{n}\}, \{\boldsymbol{\theta}_{\epsilon}\}, \{t_{\epsilon} \},\, \{\nu_{\epsilon}\},\, \{\varphi_{\epsilon}\},\, \mu,\, \Lambda \left| \{\mathbf{x}_{n}\},\, \beta_{u},\, \beta_{m},\, \lambda,\, \alpha_{0}, \gamma \right.\right),  $$

we fix *λ*=2, *α*_0_=0.3 and *γ*=0.1. The equilibrium state frequencies *β*_*u*_ and *β*_*m*_ are estimated directly from the population as the average frequencies of unmethylated and methylated states, respectively. To sample from the target distribution (11), we use a Gibbs sampler, which iteratively generates samples from the full conditional distribution of each variable of interest. The sampling procedure follows the one described in [16] with one exception. We integrated a new ‘swap clone’ move as an additional step. In this move, the parameters, assigned data and masses of two random nodes in the tree are swapped, then the structure related parameters {*ν*_*ε*_} and {*φ*_*ε*_} are resampled. The swap is accepted with a probability defined by a metropolis ratio. If, after the swap node move, the root node has no assigned data points, then more swap node moves involving the root are conducted until the root node has at least one data point assigned. In other words, we consider empty root nodes as invalid.

We used the maximum posterior expected adjusted rand (MPEAR) method from [53] to compute summary labels from MCMC samples. The method first computes the posterior similarity matrix for the labels in each MCMC sample. The posterior similarity matrix is an *N*×*N* matrix, in which each entry is the posterior probability of two data points being clustered together. Given the posterior similarity matrix, the posterior expected adjusted rand (PEAR) index can be used to assess the performance of a proposed label configuration. The labels, corresponding to the highest PEAR, are chosen as the summary cluster configuration. We used the MPEAR implementation in the R package mcclust.

At the end of each MCMC run, the reconstructed tree structure is obtained as the following. We check, for each sample, the number of clones that have weights *π*_*ε*_>0.01. We call this number the big node number. Then, all the samples can be grouped into different unique big node number categories. For each unique big node number group, we record the tree structure with the highest complete data likelihood (integrating out {*ε*_*n*_}):

(12) $$ p \left(\{\mathbf{x}_{n}\},\, \{{\boldsymbol{\theta}\epsilon}\},\{\textit{t}_{\epsilon}\}|\{\textit{v}_{\epsilon}\},\{\varphi_{\epsilon}\},\, \mu,\, \Lambda,\, \beta_{u},\, \beta_{m},\, \lambda,\, \alpha_{0},\, \gamma \right).  $$

Finally, we report the recorded tree from the most frequent unique big node number group.

### Baseline methods

We used hierarchical clustering and k-centroids as baseline clustering methods. For both methods, the R function dist with option ’binary’ was called to compute the Jaccard distance matrix of the observed sequences. The Jaccard distance matrix was then used to perform hierarchical clustering (hclust) and k-centroids (pam). To select the number of clusters, both methods were executed with a range of possible cluster numbers from 2 to 20, and the cluster number with the highest silhouette coefficient was selected. We computed silhouette coefficients with the silhouette function from the R package cluster. The coefficient is computed from the mean distances within clusters and the mean distances between clusters. It does not require the true cluster labels. The silhouette coefficient takes values between −1 and 1, with higher values indicating better clustering performance. We then estimated the methyltype sequences (methylation patterns) of each clone. For hierarchical clustering, each methyltype was computed by thresholding the mean of all sequences assigned to the clone. For k-centroids, the methyltypes were defined as the medoids. Given the estimated methyltypes, we computed minimum spanning trees from the Hamming distance matrices. We used the minimum.spanning.tree function from R package igraph. Finally, we defined the clone with the least number of methylated states to be the root clone and directed the tree accordingly. Hierarchical clustering and minimum spanning tree have been used as parts of the SPADE pipeline, which extracts a cellular hierarchy from high-dimensional cytometry data [54]. The baseline methods we used here differ from SPADE by adding a model selection step.

### Clustering performance

The clustering performance was assessed by the v-measure [51], which computes the harmonic mean of two conditional entropies, namely homogeneity and completeness. Homogeneity measures how much each cluster contains only members of a single class, while completeness measures whether all members of a given class are assigned to the same cluster. The v-measure takes values in [0, 1], with 0 and 1 indicating the worst and best clustering performance, respectively.

### Tree distance

We considered all markers that were present in the ground truth tree and in all three inferred trees. For these shared markers, we computed their pairwise shortest-path distance matrix in each tree. The lower triangular part of the distance matrices of all inferred trees were then compared to the ground truth using the sum of the absolute values of all differences between matrix entries. We named this distance measure the consensus node-based shortest path distance.

### Software and data availability

Based on the TSSB implementation [16], BitPhylogeny has been implemented in Python and R and is freely available under a GPL3 license [55]. For 2,000 data points (observed marker patterns), a single MCMC iteration takes on average about 1 s on a standard single-core computer. Additional file 2 contains an R Markdown file for reproducing all the figures in this manuscript. All data, including synthetic, methylation and single-cell data sets, are provided in the BitPhylogeny software package. The sequencing data from the single-cell study are stored in NCBI Sequence Read Archive [56] under the accession number [SRA:SRA050202].

## Additional files

Additional file 1
**Supplementary figures.** A PDF file with three supplementary figures (Figures S1, S2 and S3).

Additional file 2
**R markdown file.** A PDF file with BitPhylogeny package details and figure reproduction.

## Abbreviations

MCMC: Markov chain Monte Carlo
MPEAR: maximum posterior expected adjusted rand
PEAR: posterior expected adjusted rand
SNV: single-nucleotide variant
TSSB: tree-structured stick-breaking process
