# Supplementary material for: BitPhylogeny: a probabilistic framework for reconstructing intra-tumor phylogenies
Source: Genome Biol. 2015 Feb 13;16(1):36. doi: 10.1186/s13059-015-0592-6 (PMC4359483; doi:10.1186/s13059-015-0592-6)
Supplement: Additional file 1 — Supplementary figures. A PDF file with three supplementary figures (Figures S1, S2 and S3). [file 13059_2015_592_MOESM1_ESM.pdf]

Supplementary figures for "BitPhylogeny: A probabilistic framework for reconstructing intra-tumor phylogenies"

Ke Yuan, Thomas Sakoparnig, Florian Markowetz and Niko Beerenwinkel

November 11, 2014

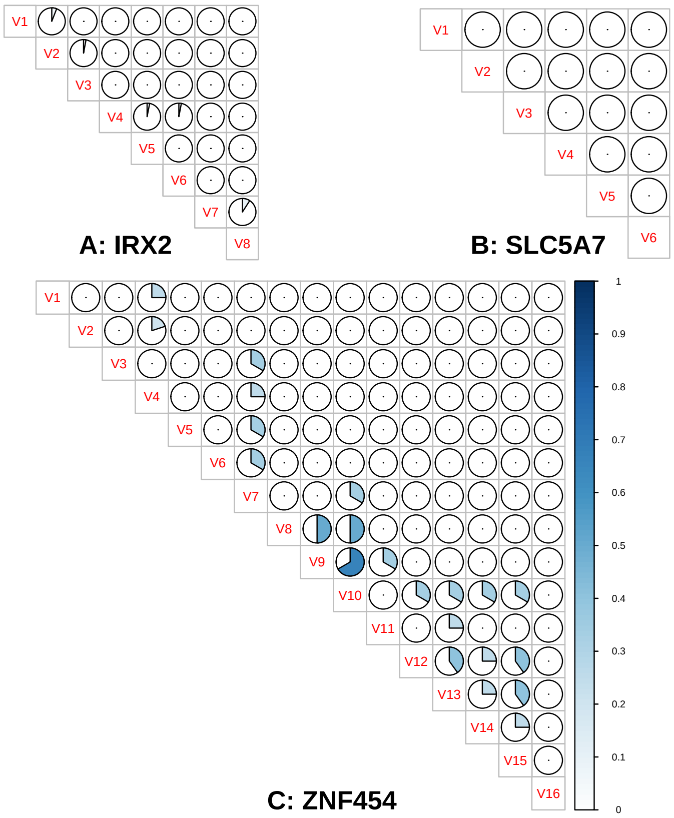

Figure S1: **Correlation profiles of three molecular clocks studied in Sottoriva et al. [1].** Percentage of significant correlation (Kendall's τ) between CpG sites among all samples. Most sites are uncorrelated.

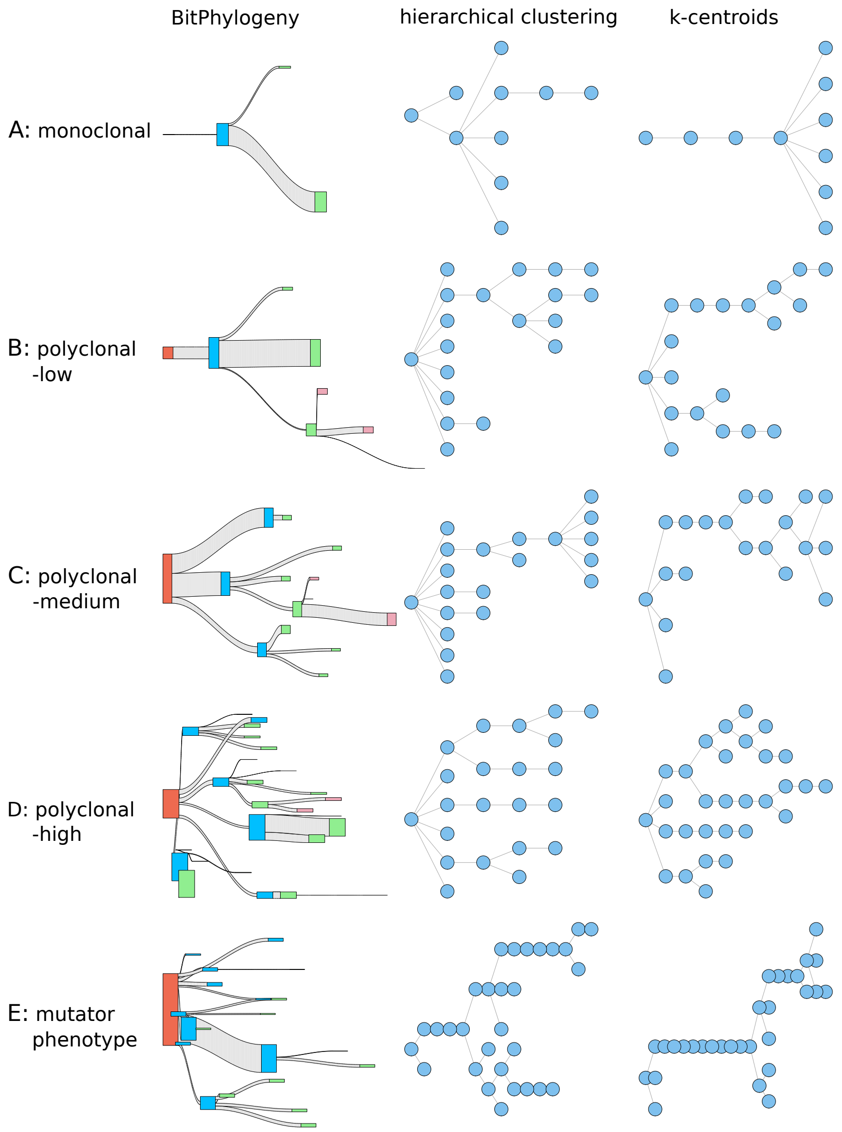

Figure S2: **Tree comparison based on datasets with** 1% **noise.** Trees shown in the same row are constructed from the same underlying true tree: **(A-E)** represent trees of monoclonal, polyclonal-(low, medium and high) and mutator phenotype. The corresponding true trees are in Figure 3 of the main text. The trees shown in the same column are constructed with the same method: `BitPhylogeny` (left), hierarchical clustering (middle) and k-centroids (right). For `BitPhylogeny` trees, the length of each edge is proportional to the branch length between the two clones. For each clone, the in-edge width is proportional to the number of associated reads.

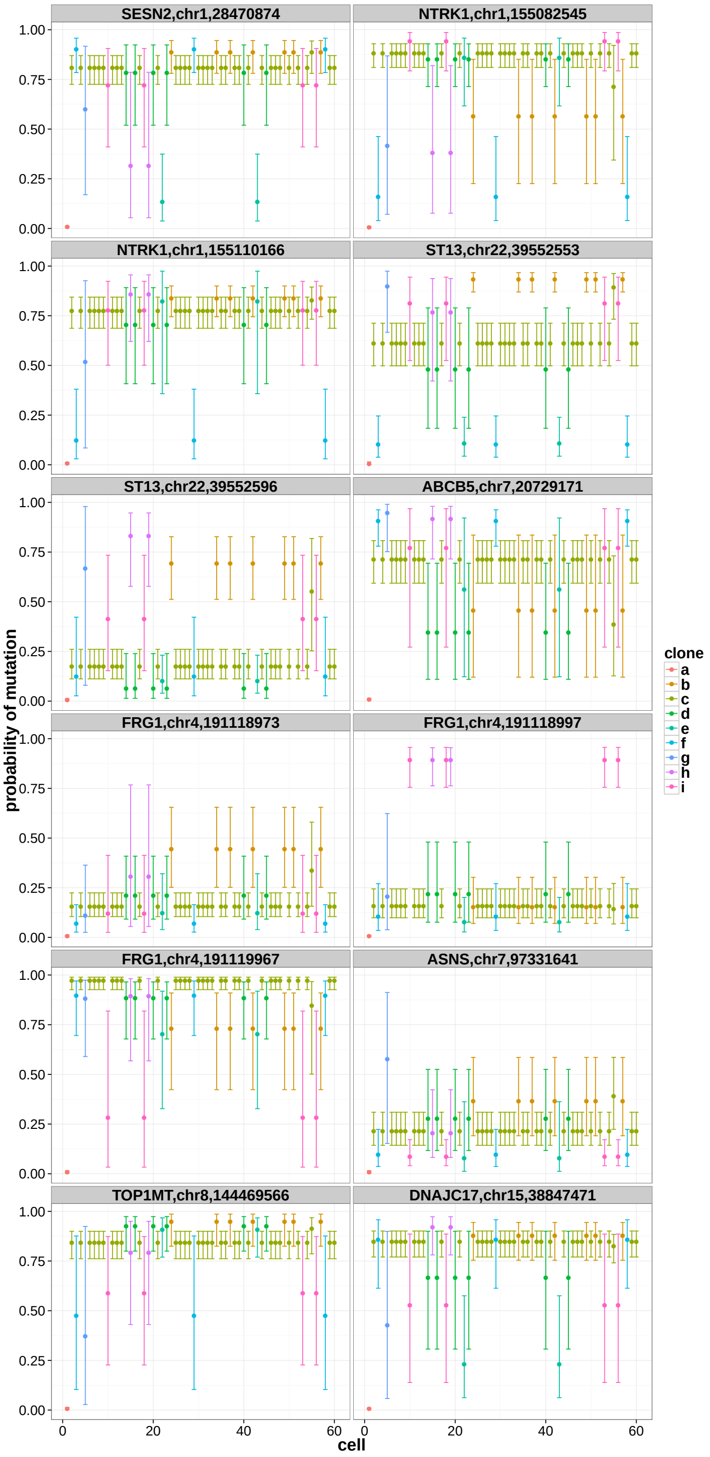

Figure S3: **Estimated genotype profiles of eight key genes.** Probability of mutation of 12 SNVs located in eight genes across all cells. The error bars summarize 50,000 MCMC samples and are colour-coded according to clone membership.

References

[1] Sottoriva, A., Spiteri, I., Shibata, D., Curtis, C., and Tavaré, S. (2013). Single-molecule genomic data delineate patient-specific tumor profiles and cancer stem cell organization. *Cancer Res*, 73(1):41–49.
