# Supplementary material for: BitPhylogeny: a probabilistic framework for reconstructing intra-tumor phylogenies
Source: Genome Biol. 2015 Feb 13;16(1):36. doi: 10.1186/s13059-015-0592-6 (PMC4359483; doi:10.1186/s13059-015-0592-6)

R markdown file for "BitPhylogeny: A probabilistic framework for reconstructing intra-tumor phylogenies"

Contents

1 The `BitPhylogeny` package **1**

1.1 Installation . . . 1

1.2 An example . . . 2

2 Reproducing V-measures in Figure 3 **9**

3 Reproducing number of nodes and maximum tree depth plots in Figure 3 **10**

4 Reproducing tree distance in Figure 4 **12**

5 Reproducing Figure 5 **15**

6 Reproducing Figure 6 **17**

7 Reproducing Figure 7 and S3 **20**

8 Reproducing Figure S1 **24**

1 The `BitPhylogeny` package

1.1 Installation

The `BitPhylogeny` package depends on several `python` and `R` packages. The first step is to make sure the following the packages are installed. * `python`: numpy, scipy, scikit-learn: http://scikit-learn.org/stable/, rpy2: http://rpy.sourceforge.net/, pandas: http://pandas.pydata.org/, h5py: http://www.h5py.org/. * `R`: rPython, mcclust, e1071, igraph, gplots, riverplot.

Secondly, clone the BitPhylogeny repository

```
git clone git@bitbucket.org:ke_yuan/bitphylogeny.git
cd bitphylogeny
```

The third step is install the BitPhylogeny python package. To do this, navigate into the python directory and run the following

```
cd python
sudo python setup.py install
```

Finally, install the R package

```
cd ../R
R CMD INSTALL bitphylogenyR_0.1.tar.gz
```

1.2 An example

1.2.1 BitPhylogeny

We use an example dataset

```
library('bitphylogenyR')
```

```
## Loading required package: rPython
## Loading required package: RJSONIO
## Loading required package: igraph
```

```
example_file <- system.file('sample_data.csv', package='bitphylogenyR')
tmp <- read.csv( example_file )
head(tmp)
```

```
##   V1 V2 V3 V4 V5 V6 V7 V8 V9
## 1  0  0  0  0  0  0  0  0  1
## 2  0  0  0  0  0  0  0  0  1
## 3  0  0  0  0  0  0  0  0  1
## 4  0  0  0  0  0  0  0  0  1
## 5  0  0  0  0  0  0  0  0  1
## 6  0  0  0  0  0  0  0  0  1
```

Note that the last column is set to be the true cluster label of each data point. We separate the data and its label.

```
x <- tmp[,-dim(tmp)[2]]
true_label <- tmp[,dim(tmp)[2]]
```

Run the BitPhylogeny analysis as the following

```
bitphyloR(example_file, './output', T, 200, 50, 5)
```

```
## NULL
```

By default, `bitphyloR` runs with methylation model setting. To analyse mutation data, one can use the `mode` parameter

```
bitphyloR(example_file, './output', T, 200, 50, 5, mode = "mutation")
```

The program saves the results in the directory 'output'.

```
dir('./output', recursive=T)
```

```
##  [1] "sample_data.csv/mcmc-traces/branch_traces.csv"
##  [2] "sample_data.csv/mcmc-traces/label_traces.csv"
##  [3] "sample_data.csv/mcmc-traces/mpear_label.csv"
##  [4] "sample_data.csv/mcmc-traces/mpear_vmeasure"
##  [5] "sample_data.csv/mcmc-traces/mpear_vmeasure.csv"
```

```
##  [6] "sample_data.csv/mcmc-traces/node_depth_traces.csv"
##  [7] "sample_data.csv/mcmc-traces/other_traces.csv"
##  [8] "sample_data.csv/mcmc-traces/params_traces/array_0.npz"
##  [9] "sample_data.csv/mcmc-traces/params_traces/array_1.npz"
## [10] "sample_data.csv/mcmc-traces/params_traces/array_2.npz"
## [11] "sample_data.csv/mcmc-traces/params_traces/array_3.npz"
## [12] "sample_data.csv/mcmc-traces/params_traces/array_4.npz"
## [13] "sample_data.csv/mcmc-traces/params_traces/array_5.npz"
## [14] "sample_data.csv/mcmc-traces/params_traces/array_6.npz"
## [15] "sample_data.csv/mcmc-traces/params_traces/array_7.npz"
## [16] "sample_data.csv/mcmc-traces/params_traces/array_8.npz"
## [17] "sample_data.csv/mcmc-traces/params_traces/array_9.npz"
## [18] "sample_data.csv/mcmc-traces/params_traces.h5"
## [19] "sample_data.csv/mcmc-traces/root_param_traces.csv"
## [20] "sample_data.csv/mcmc-traces/vmeasure_traces.csv"
## [21] "sample_data.csv/treescripts/nodes-3.gdl"
## [22] "sample_data.csv/treescripts/nodes-4.gdl"
## [23] "sample_data.csv/treescripts/nodes-5.gdl"
## [24] "sample_data.csv/treescripts/nodes-6.gdl"
## [25] "sample_data.csv/treescripts/tree-freq.csv"
```

The clustering performance is assessed by the V-measure. In addition, the label trace is summarised by the maximum posterior expected adjusted Rand method.

```
compute_vmeasures('./output/sample_data.csv',
                  system.file('sample_data.csv', package='bitphylogenyR'))
```

We can visualise the results in the following way

```
fp <- get_path('./output/sample_data.csv', 'mcmc-traces')
vmeasure_traces <- as.matrix(load_vmeasures(fp, 'vmeasure_traces.csv'))
mpear_vmeasure <- as.matrix(load_vmeasures(fp, 'mpear_vmeasure.csv'))
class(vmeasure_traces) <- 'numeric'
class(mpear_vmeasure) <- 'numeric'
par(cex.lab=1.5, cex.axis=1.5)
boxplot(vmeasure_traces, outline=F, cex.main=1.3, ylim=c(0,1),
        border=c('gray60'), col='gray90')
points(c(1,2,3),mpear_vmeasure, pch=22,cex = 1.5, bg= 'black')
colors1 <- c("gray90",'black')
colors2 <- c("gray",'black')
add_legend("bottomleft", legend=c("traces", 'BitPhylogeny'),
           pch=c(22,22), inset = c(0.1,0.20), col=colors1,
           pt.bg=colors2,
           horiz=F, bty='n', cex=1.5)
```

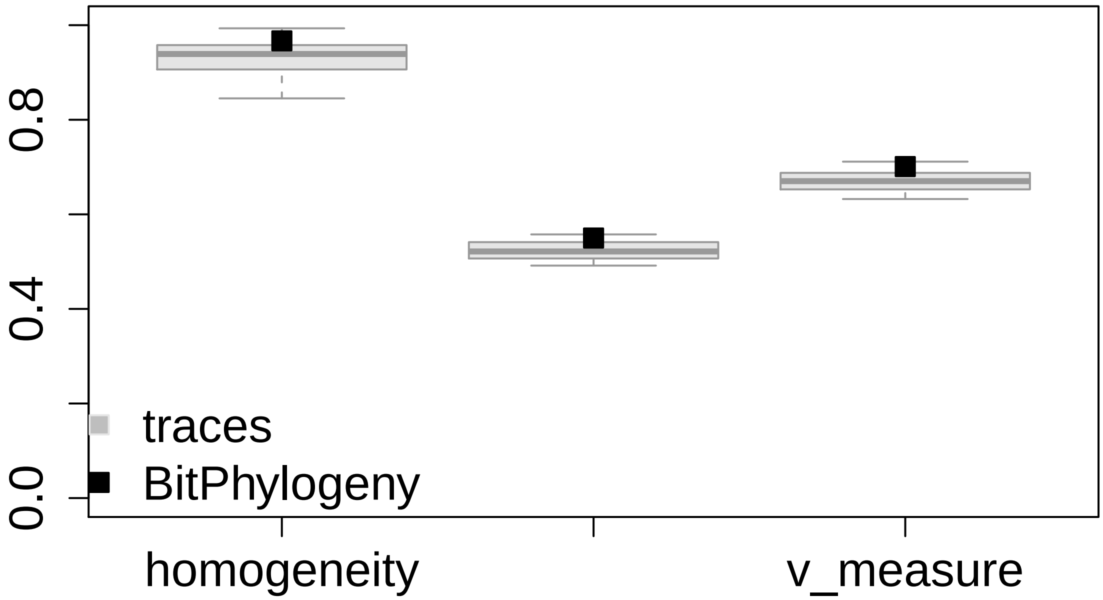

The resulting trees are stored in the `treescripts` directory. The file `tree-freq` contains the appearance frequency of each tree in the folder.

```
treefreq <- read.csv('./output/sample_data.csv/treescripts//tree-freq.csv')
treefreq
```

```
##   unique_node_num  freq
## 1               3 0.325
## 2               4 0.550
## 3               5 0.100
## 4               6 0.025
```

```
plot_sankey_mft('./output/sample_data.csv/treescripts//tree-freq.csv')
```

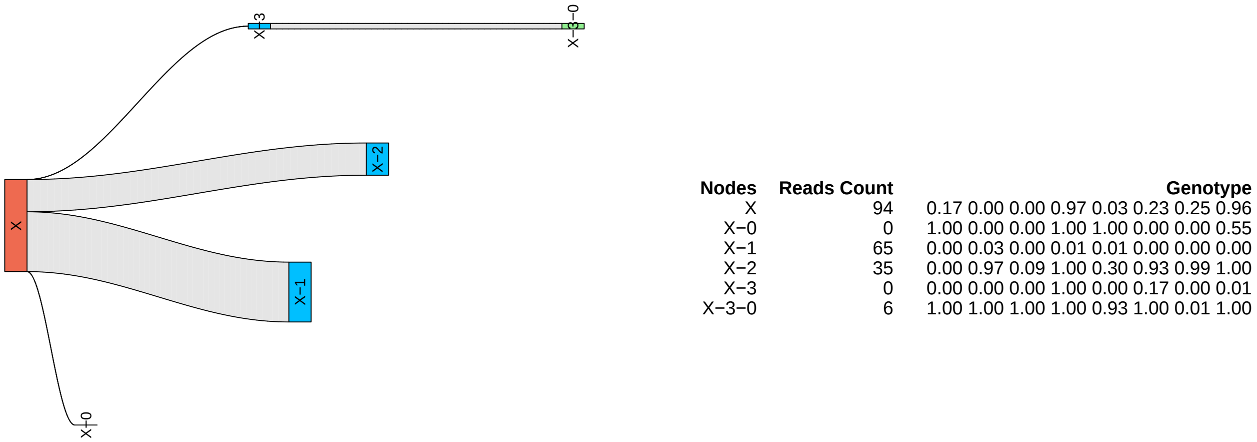

Alternatively, the .gdl files can be visualised with the aisee3 software.

1.2.2 Baseline methods

The baseline methods are consisted of clustering followed by minimum spanning tree construction.

1.2.2.1 Hierarchical clustering

We first compute the Jacard distance matrix for the sequences.

```
dis <- dist(x, 'binary')
```

Then, we use the R function `hclust` to perform hierarchical clustering based on the previously computed distance matrix.

```
hc <- hclust(dis)
hc
```

```
##
## Call:
## hclust(d = dis)
##
## Cluster method   : complete
## Distance         : binary
## Number of objects: 200
```

The resulting labels of each data point is obtained with the function `cutree`. The function takes the output of `hclust` and the number of desired clusters `k`. In this case, we set `k` to be 7.

```
label <- cutree(hc, k=7)
label
```

```
##   [1] 1 1 1 1 1 1 1 1 1 1 1 1 1 1 1 1 1 1 1 1 1 1 1 1 1 1 1 2 1 1 1 1 1 1 1
##  [36] 1 1 1 3 1 1 1 1 1 1 1 1 3 1 1 1 1 1 1 1 1 1 1 1 1 1 1 1 1 1 4 4 4 4 4
##  [71] 4 4 4 4 4 4 4 4 4 4 4 4 4 4 4 4 4 4 4 5 4 4 4 4 4 4 4 4 4 6 4 4 4 4 4
## [106] 6 4 4 4 4 4 4 4 6 4 4 4 4 4 5 4 4 4 4 4 4 4 4 4 4 4 4 4 4 4 4 4 4 4 4
## [141] 4 4 4 4 4 4 4 4 4 4 4 4 7 4 4 4 4 4 5 5 5 5 5 5 5 5 5 5 5 5 5 5 7 7 7
## [176] 7 7 7 6 6 6 6 6 5 5 5 5 5 7 7 7 7 7 7 4 4 7 7 7 7
```

When there is a range of cluster number hypothesis, we compute a list of possible labels.

```
K <- seq(2,14,1)
hc_cand <- lapply(K, function(ii) cutree(hc, ii) )
```

Each of these hypothesis is evaluated by the Silhouette score. The one with the highest score is chosen as the clustering result.

```
library(cluster)
hc_silhouette_res <- sapply(1:length(K),
                              function(ii)
                                summary( silhouette(hc_cand[[ii]] ,dis) )$avg.width )
idx <- which.max( hc_silhouette_res )
hc_label <- hc_cand[[idx]]
hc_label
```

```
##   [1]  1  1  1  1  1  1  1  1  1  1  1  1  1  1  1  1  1  1  1  1  1  1  1
##  [24]  1  1  1  1  2  1  1  1  1  1  1  1  1  1  1  3  1  1  1  1  1  1  1
##  [47]  1  3  1  1  1  1  1  1  1  1  1  1  1  1  1  1  1  1  1  4  4  4  5
##  [70]  4  4  4  4  4  4  4  4  4  4  4  4  4  4  4  6  6  6  6  6  7  6  6
```

```
## [93]   6  6  6  6  6  6  6  8  6  6  6  6  6  8  6  6  6  6  6  6  6  9  6
## [116]  6  6  6  6 10  6  6  6  6  6  6  6  4  4  4  4  4  4  4  4  5  5  5
## [139]  5  5  5  5  5  5  5  5  5  5  5  5  5  5 11  5  5  5  5  5  7  7  7
## [162]  7  7  7  7 12  7  7  7  7  7  7 11 11 11 11 11 11  9  9  9  9  9 13
## [185] 13 13 13 13 14 14 14 14 14 14  4  5 11 11 11 14
```

Once the label is computed, we compute the genotype of each cluster as the following

```
clone <- sapply(unique(hc_label), function(i) which(hc_label==i) )
n <- length(clone)
hc_genotype <- matrix(0, n, dim(x)[2])
for (i in 1:n){
    idx <- clone[[i]]
    if ( length(idx)==1 ){
      hc_genotype[i,] <- as.matrix(x[idx,])
    }else{
      hc_genotype[i,] <- as.numeric( colMeans(as.matrix(x[idx,])) > 0.5 )
    }
}
hc_genotype
```

```
##       [,1] [,2] [,3] [,4] [,5] [,6] [,7] [,8]
##  [1,]    0    0    0    0    0    0    0    0
##  [2,]    0    0    0    0    1    0    0    0
##  [3,]    0    1    0    0    0    0    0    0
##  [4,]    0    0    0    1    0    0    1    1
##  [5,]    0    1    0    1    0    1    1    1
##  [6,]    0    0    0    1    0    0    0    1
##  [7,]    0    0    0    1    0    1    0    1
##  [8,]    0    0    0    1    0    0    0    0
##  [9,]    1    0    0    1    0    0    0    1
## [10,]    0    0    0    0    0    0    0    1
## [11,]    0    1    0    1    1    1    1    1
## [12,]    0    0    0    0    0    1    0    1
## [13,]    0    0    0    1    1    1    0    1
## [14,]    1    1    1    1    1    1    0    1
```

Finally, we put the above steps into a function which gives the label and genotype estimates.

```
get_label_hc
```

```
## function (x, K)
## {
##     dis <- dist(x, "binary")
##     hc_cand <- lapply(K, function(ii) cutree(hclust(dis), ii))
##     hc_silhouette_res <- sapply(1:length(K), function(ii) summary(silhouette(hc_cand[[ii]],
##         dis))$avg.width)
##     idx <- which.max(hc_silhouette_res)
##     hc_label <- hc_cand[[idx]]
##     clone <- sapply(unique(hc_label), function(i) which(hc_label ==
##         i))
##     n <- length(clone)
```

```
##     genotype <- matrix(0, n, dim(x)[2])
##     for (i in 1:n) {
##         idx <- clone[[i]]
##         if (length(idx) == 1) {
##             genotype[i, ] = as.matrix(x[idx, ])
##         }
##         else {
##             genotype[i, ] = as.numeric(colMeans(as.matrix(x[idx,
##                 ])) > 0.5)
##         }
##     }
##     return(list(label = hc_label, genotype = genotype))
## }
## <environment: namespace:bitphylogenyR>
```

1.2.2.2 K-centroids clustering The k-centroids methods uses the same distance matrix compute above.

```
kc = pam(dis, 7)
kc
```

```
## Medoids:
##       ID
## [1,]  65  65
## [2,] 194 194
## [3,] 158 158
## [4,] 195 195
## [5,] 127 127
## [6,] 172 172
## [7,] 135 135
## Clustering vector:
##   [1] 1 1 1 1 1 1 1 1 1 1 1 1 1 1 1 1 1 1 1 1 1 1 1 1 1 1 1 2 1 1 1 1 1 1 1
##  [36] 1 1 1 3 1 1 1 1 1 1 1 1 3 1 1 1 1 1 1 1 1 1 1 1 1 1 1 1 1 1 1 4 4 4 3 4
##  [71] 4 4 4 4 4 4 4 4 4 4 4 4 4 4 5 5 5 5 5 6 5 5 5 5 5 5 5 5 5 5 5 5 5 5 5
## [106] 5 5 5 5 5 5 5 5 7 5 5 5 5 5 5 5 5 5 5 5 5 5 7 7 7 7 7 7 7 7 3 3 3 3 3
## [141] 3 3 3 3 3 3 3 3 3 3 3 3 3 3 3 3 3 3 3 6 6 6 6 6 6 6 6 6 6 6 6 3 3 3
## [176] 3 3 3 7 7 7 7 7 6 6 6 6 6 2 2 2 2 2 2 2 4 3 2 2 2 2
## Objective function:
##   build    swap
## 0.05225 0.05225
##
## Available components:
## [1] "medoids"    "id.med"     "clustering" "objective"  "isolation"
## [6] "clusinfo"   "silinfo"    "diss"       "call"
```

In this case, the genotypes can be obtained as the metroids. The Silhouette score is used to choose the number of clusters.

```
kc_cand <- lapply(K, function(ii) pam( dis, ii) )
kc_silhouette_res <- sapply(1:length(K), function(ii)
                              summary( silhouette(kc_cand[[ii]]$clustering,dis) )$avg.width )
idx <- which.max( kc_silhouette_res )
```

```
kc_label <- kc_cand[[idx]]$clustering
kc_label
```

```
##   [1]  1  1  1  1  1  1  1  1  1  1  1  1  1  1  1  1  1  1  1  1  1  1  1
##  [24]  1  1  1  1  2  1  1  1  1  1  1  1  1  1  1  3  1  1  1  1  1  1  1
##  [47]  1  3  1  1  1  1  1  1  1  1  1  1  1  1  1  1  1  1  1  4  4  4  5
##  [70]  4  4  4  4  4  4  4  4  4  4  4  4  4  4  4  6  6  6  6  6  7  6  6
##  [93]  6  6  6  6  6  6  6  8  6  6  6  6  6  8  6  6  6  6  6  6  6  9  6
## [116]  6  6  6  6 10  6  6  6  6  6  6  6 11 11 11 11 11 11 11 11  5  5  5
## [139]  5  5  5  5  5  5  5  5  5  5  5  5  5  5 12  5  5  5  5  5  5  7  7
## [162]  7  7  7  7  7  7  7  7  7  7  7 12 12 12 12 12 12  9  9  9  9  9 13
## [185] 13 13 13 13 14 14 14 14 14 14  4  5 12 12 12 14
```

```
kc_genotype <- x[kc_cand[[idx]]$medoids,]
kc_genotype
```

```
##     V1 V2 V3 V4 V5 V6 V7 V8
## 65   0  0  0  0  0  0  0  0
## 28   0  0  0  0  1  0  0  0
## 48   0  1  0  0  0  0  0  0
## 195  0  0  0  1  0  0  1  1
## 158  0  1  0  1  0  1  1  1
## 127  0  0  0  1  0  0  0  1
## 172  0  0  0  1  0  1  0  1
## 106  0  0  0  1  0  0  0  0
## 183  1  0  0  1  0  0  0  1
## 120  0  0  0  0  0  0  0  1
## 135  1  0  0  1  0  0  1  1
## 178  0  1  0  1  1  1  1  1
## 188  0  0  0  1  1  1  0  1
## 194  1  1  1  1  1  1  0  1
```

We also wrapped up a function for k-centroids clustering.

```
get_label_kc
```

```
## function (x, K)
## {
##     dis <- dist(x, "binary")
##     kc_cand <- lapply(K, function(ii) pam(dis, ii))
##     kc_silhouette_res <- sapply(1:length(K), function(ii) summary(silhouette(kc_cand[[ii]]$clustering
##         dis))$avg.width)
##     idx <- which.max(kc_silhouette_res)
##     kc_label <- kc_cand[[idx]]$clustering
##     kc_genotype <- x[kc_cand[[idx]]$medoids, ]
##     return(list(label = kc_label, genotype = kc_genotype))
## }
## <environment: namespace:bitphylogenyR>
```

1.2.3 Tree building

We construct the minimum spanning tree based the clustering results from the previous stage.

```r
mst <- get_mst(hc_genotype)
plot_mst(hc_genotype, hc_label, mst, flag=F)
```

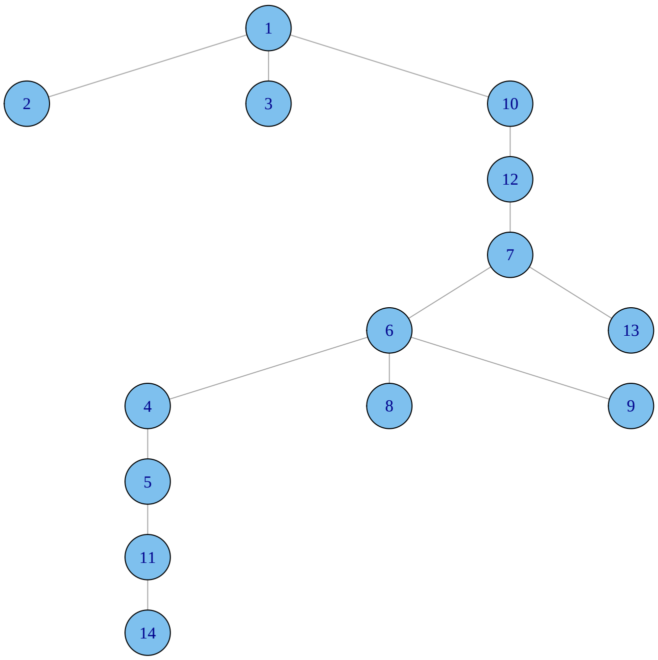

Nodes	Read Counts	Genotype
1	62	0 0 0 0 0 0 0 0
2	1	0 0 0 0 1 0 0 0
3	2	0 1 0 0 0 0 0 0
4	27	0 0 0 1 0 0 1 1
5	24	0 1 0 1 0 1 1 1
6	38	0 0 0 1 0 0 0 1
7	14	0 0 0 1 0 1 0 1
8	2	0 0 0 1 0 0 0 0
9	6	1 0 0 1 0 0 0 1
10	1	0 0 0 0 0 0 0 1
11	10	0 1 0 1 1 1 1 1
12	1	0 0 0 0 0 1 0 1
13	5	0 0 0 1 1 1 0 1
14	7	1 1 1 1 1 1 0 1

2 Reproducing V-measures in Figure 3

```r
data(saved_vmeasures)

mcmc_vmeasures <- saved_vmeasures$mcmc_vmeasures
hc_vmeasures <- saved_vmeasures$hc_vmeasures
kc_vmeasures <- saved_vmeasures$kc_vmeasures
mpear_vmeasures <- saved_vmeasures$mpear_vmeasures

par(mfrow=c(1,2), oma = c(3,3,0,0) + 0.1,
    mar = c(0,0,1,0.5) + 0.1, cex.lab=1.5, cex.axis=1.5)
boxplot(mcmc_vmeasures$big_clone, outline=F,
        ylim=c(0.5,1) ,
        cex.main=1.3,
        border=c('gray60'), col='gray90')
points( mpear_vmeasures$big_clone, pch=22,cex = 1.5, bg= 'black')
points( hc_vmeasures$big_clone, pch=24,cex = 1.5, bg= 'black')
points( kc_vmeasures$big_clone, pch=25,cex = 1.5, bg= 'black')

boxplot(mcmc_vmeasures$small_clone, outline=F,
        ylim=c(0.5,1) ,
        yaxt='n',cex.main=1.3,
        border=c('gray60'), col='gray90')
points( mpear_vmeasures$small_clone, pch=22, cex = 1.5, bg= 'black')
points( hc_vmeasures$small_clone, pch=24, cex=1.5, bg= 'black')
points( kc_vmeasures$small_clone, pch=25, cex = 1.5, bg= 'black')
```

```
colors1 <- c("gray90",'black', 'black', "black")
colors2 <- c("gray",'black', 'black', "black")
add_legend("bottomleft", legend=c("traces", 'BitPhylogeny',
                               'k-centroids',
                               'hierarchical clustering'),
           pch=c(22,22,25,24), inset = c(0.1,0.13), col=colors1,
           pt.bg=colors2,
           horiz=F, bty='n', cex=1.5)
title(xlab = "error",
      ylab = "v-measure",
      outer = TRUE, line = 2.2)
```

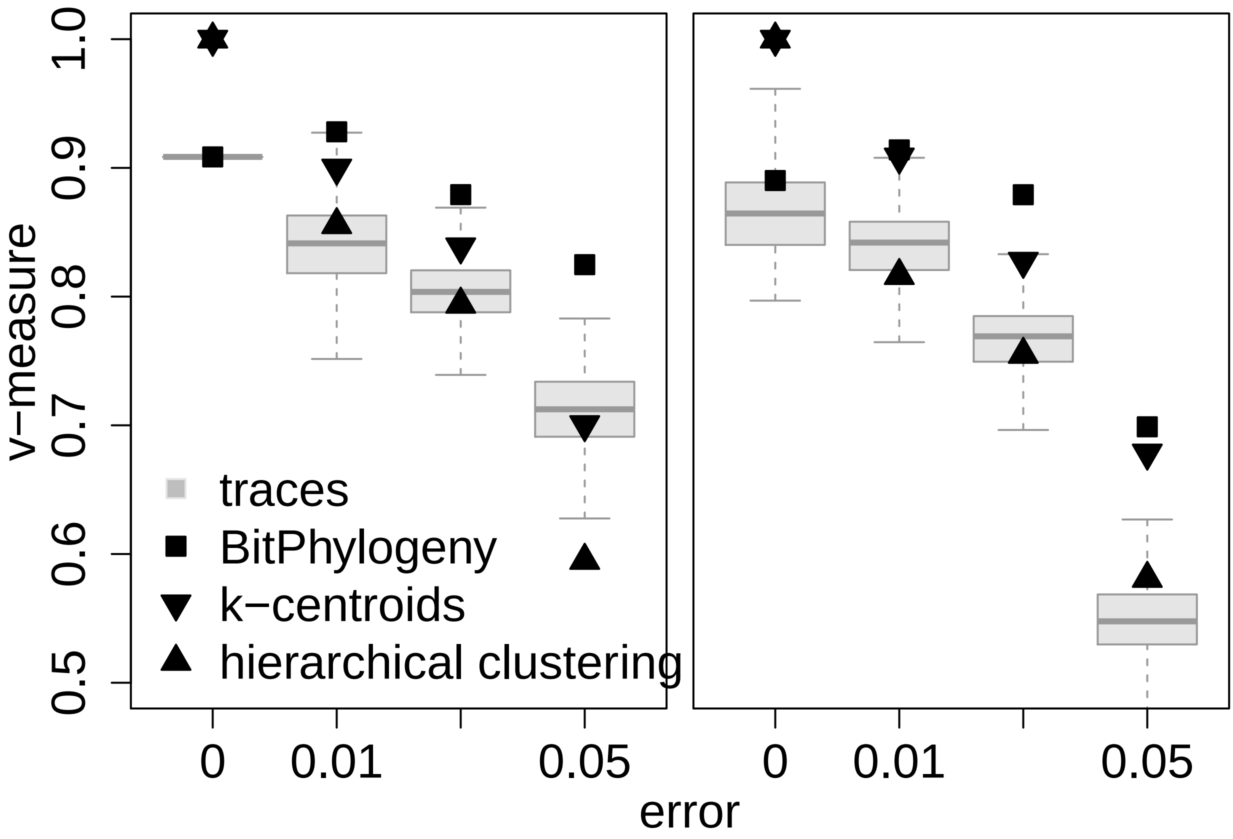

3 Reproducing number of nodes and maximum tree depth plots in Figure 3

```
big_clone_t <- c(3, 7)
big_clone_bit <- c(2, 5)
big_clone_hc <- c(4, 7)
big_clone_kc <- c(4, 7)
big_clone_bit <- rbind(big_clone_bit,
                      c(3, 7), c(2,9 ), c(2,7) )
big_clone_hc <- rbind(big_clone_hc,
                     c(5, 19), c(5,20), c(6,20))
```

```r
big_clone_kc <- rbind(big_clone_kc,
                     c(5, 20), c(5, 20), c(5,20))

small_clone_t <- c(4, 12)
small_clone_bit <- c(2, 8)
small_clone_hc <- c(5, 12)
small_clone_kc <- c(5, 12)

small_clone_bit <- rbind(small_clone_bit,
                        c(3, 14),c(2,16 ), c(2,13) )
small_clone_hc <- rbind(small_clone_hc,
                       c(7, 19),c(5,20), c(9,20))
small_clone_kc <- rbind(small_clone_kc,
                       c(9, 20),c(7, 20), c(6,20))

par(mfrow=c(1,2), oma = c(3,3,0,0) + 0.1,
    mar = c(0,0,1,0.5) + 0.1, cex.lab=1.5, cex.axis=1.5)
color <- c('blue', 'red', 'red', 'red')
plot(big_clone_t[2], big_clone_t[1],pch=3, ylim=c(0,10) ,xlim= c(5,22)
     ,cex=1.5)
points(big_clone_bit[,2], big_clone_bit[,1],pch=0,cex=1.5, col=color)
points(big_clone_hc[,2], big_clone_hc[,1],pch=2,cex=1.5, col=color)
points(big_clone_kc[,2], big_clone_kc[,1],pch=6,cex=1.5, col=color)

plot(small_clone_t[2], small_clone_t[1], pch=3, ylim=c(0,10),
     xlim= c(5,22), cex=1.5, yaxt='n')
points(small_clone_bit[,2], small_clone_bit[,1], pch=0, cex=1.5, col=color)
points(small_clone_hc[,2], small_clone_hc[,1], pch=2, cex=1.5, col=color)
points(small_clone_kc[,2], small_clone_kc[,1], pch=6, cex=1.5, col=color)

colors1 <- c("black",'black', 'black', "black")
colors2 <- c("black",'black', 'black', "black")
add_legend("topleft", legend=c("truth", 'BitPhylogeny',
                                  'k-centroids',
                                  'hierarchical clustering'),
           pch=c(3,0,6,2), inset = c(0.08,0.02), col=colors1,
           pt.bg=colors2,
           horiz=F, bty='n', cex=1.5)
add_legend("topleft", legend=c("noiseless", 'noise levels: \n0.01,0.02,0.05'),
           inset = c(0.50,0.02), text.col=c('blue','red'),
           horiz=F, bty='n', cex=1.5)
title(xlab = "number of clones",
      ylab = "maximum tree depth",
      outer = TRUE, line = 2.2)
```

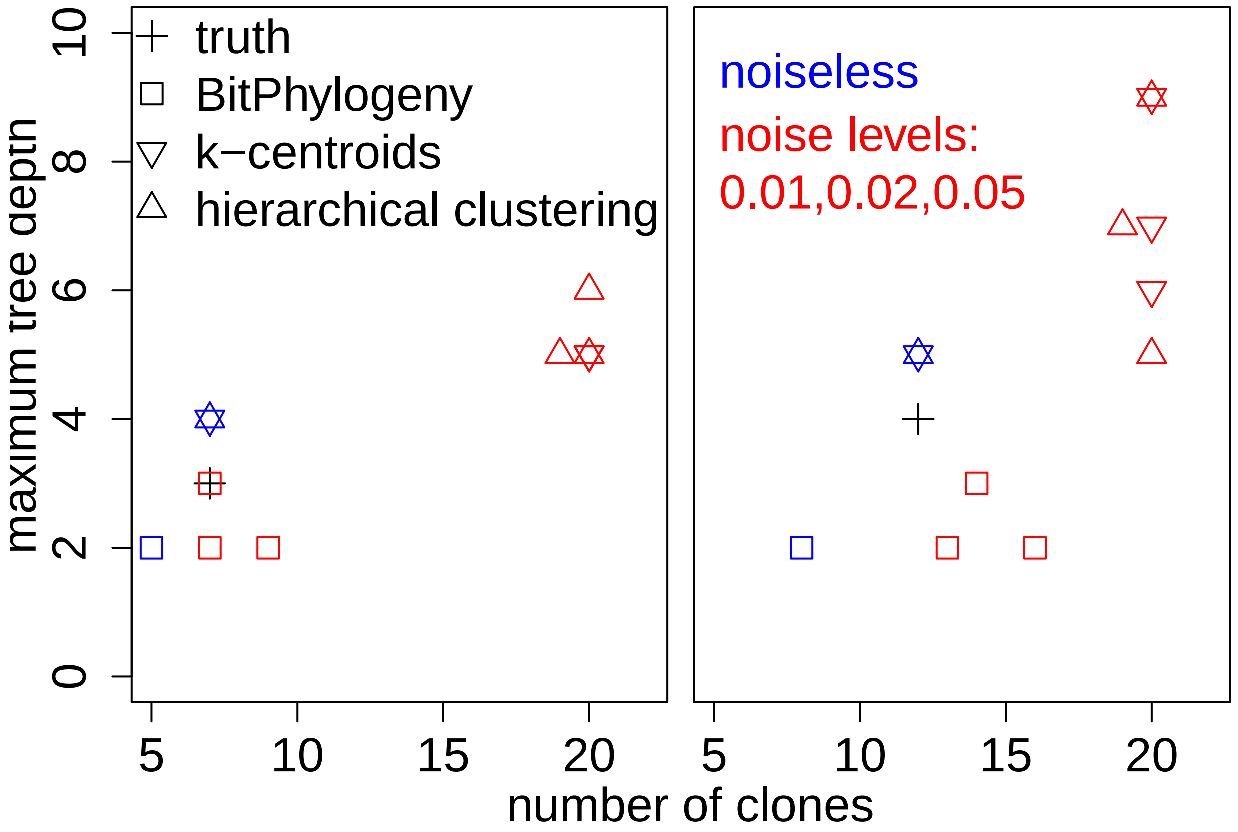

4 Reproducing tree distance in Figure 4

```
library(ggplot2)
library(plyr)
library(grid)


metrics1 <- get_common_tree_distance_df("/media/nas/BitPhylogeny//methylation/mono_tree_simulations",
                                        "/media/nas/BitPhylogeny//methylation/mono_tree_baseline",
                                        "/media/nas/BitPhylogeny//methylation/mono_tree_baseline",
                                        "mono-clone",
                                        "/media/nas/BitPhylogeny//methylation/tree-truth-full-mono-methy

metrics2 <- get_common_tree_distance_df("/media/nas/BitPhylogeny//methylation/big_tree_simulations",
                                        "/media/nas/BitPhylogeny//methylation/big_tree_baseline",
                                        "/media/nas/BitPhylogeny//methylation/big_tree_baseline",
                                        "big-clone",
                                        "/media/nas/BitPhylogeny//methylation/tree-truth-full-big-methy

metrics3 <- get_common_tree_distance_df("/media/nas/BitPhylogeny//methylation/small_tree_simulations",
                                        "/media/nas/BitPhylogeny//methylation/small_tree_baseline",
                                        "/media/nas/BitPhylogeny//methylation/small_tree_baseline",
                                        "small-clone",
                                        "/media/nas/BitPhylogeny//methylation/tree-truth-full-small-met
```

```
metrics4 <- get_common_tree_distance_df("/media/nas/BitPhylogeny//methylation/hyper_tree_simulations",
                                         "/media/nas/BitPhylogeny//methylation/hyper_tree_baseline",
                                         "/media/nas/BitPhylogeny//methylation/hyper_tree_baseline",
                                         "hyper-clone",
                                         "/media/nas/BitPhylogeny//methylation/tree-truth-full-hyper-met


metrics5 <- get_common_tree_distance_df("/media/nas/BitPhylogeny//methylation/star_tree_simulations",
                                         "/media/nas/BitPhylogeny//methylation/star_tree_baseline",
                                         "/media/nas/BitPhylogeny//methylation/star_tree_baseline",
                                         "star-clone",
                                         "/media/nas/BitPhylogeny//methylation/tree-truth-full-star-meth

metrics <- rbind(metrics1, metrics2, metrics3, metrics4, metrics5)
levels(metrics$tree_type) <- c("monoclonal",
                               "polyclonal-L",
                               "polyclonal-M",
                               "polyclonal-H",
                               "mutator")
levels(metrics1$tree_type) <- c("monoclonal",
                                "polyclonal-L",
                                "polyclonal-M",
                                "polyclonal-H",
                                "mutator")

print(ggplot(data = metrics, aes(x = tree_type, y = distance,  color = method,
                                 shape = method)) +
        geom_jitter( position = position_dodge(width = 0.75), alpha = 0.8,
                     size = 5 ) + geom_boxplot(fill = NA, outlier.colour = NA) +
        theme_bw() + xlab("tree type") +
        theme(axis.title.x = element_text(face="bold", size=25),
              axis.text.x  = element_text(vjust=0.5, size=22, angle = 0),
              axis.title.y = element_text(face="bold", size=25),
              axis.text.y  = element_text(size=20),
              legend.title = element_blank(),
              legend.text = element_text(size = 22),
              legend.position = c(0.2,0.8),
              legend.key.size = unit(1.5, "cm")))
```

```
## ymax not defined: adjusting position using y instead
```

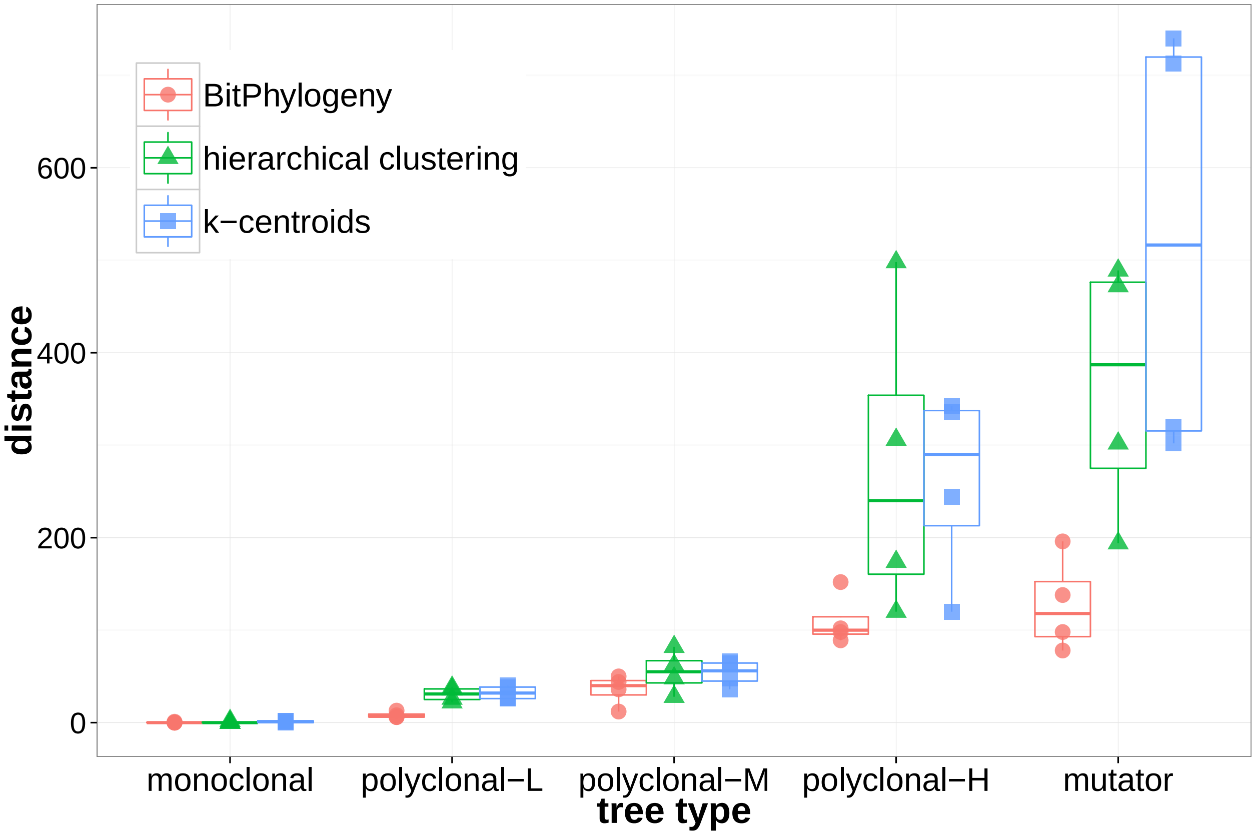


```
print(ggplot(data = metrics1, aes(x = tree_type, y = distance,  color = method,
                                  shape = method)) +
        geom_jitter( position = position_dodge(width = 0.75), alpha = 0.8,
                     size = 5) + geom_boxplot(fill = NA, outlier.colour = NA) +
        theme_bw() +  xlab("tree type") +
      theme(axis.title.x = element_blank(),
            axis.title.y = element_blank(),
            axis.text.x  = element_text(vjust=0.5, size=22),
            axis.text.y  = element_text(size=20),
            legend.position = "none"))
```

```
## ymax not defined: adjusting position using y instead
```

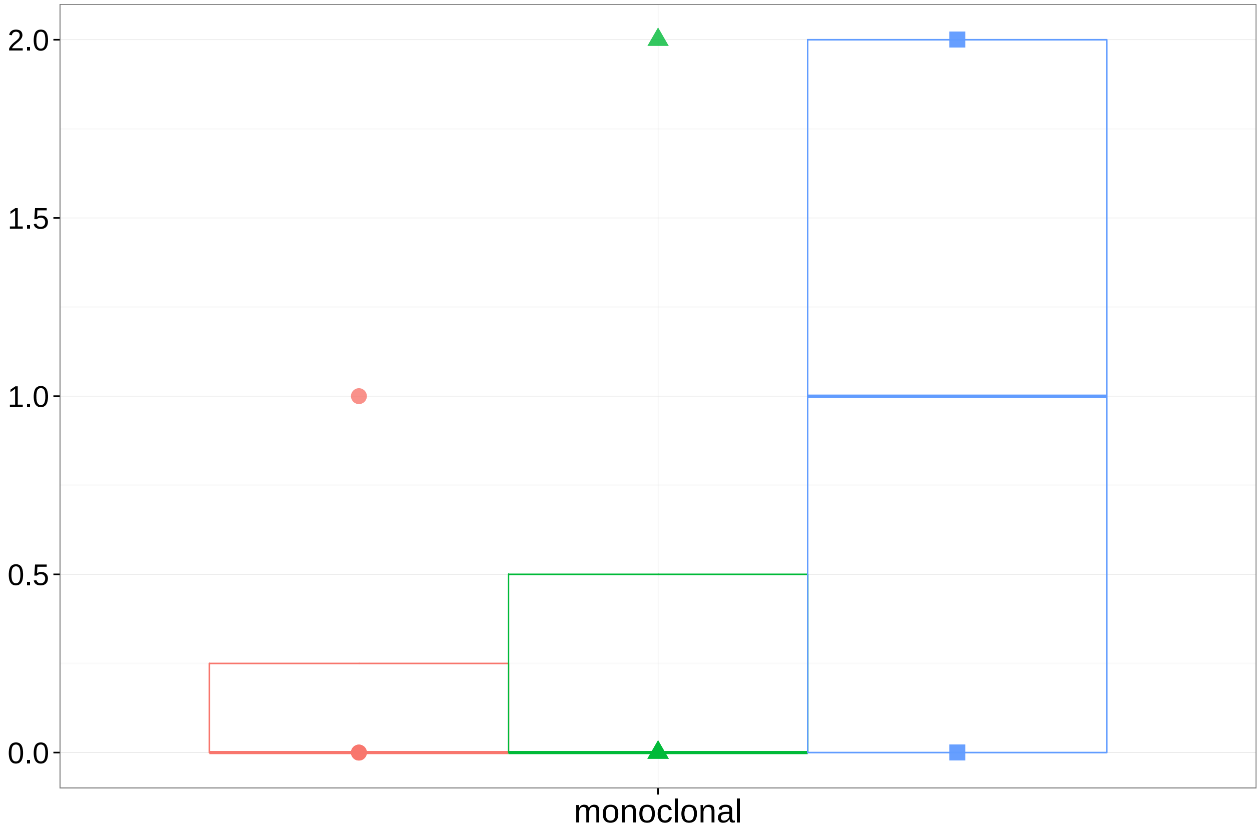

5 Reproducing Figure 5

```
CT_R1<-read.table("/media/nas/BitPhylogeny/methylation/backup/final_mcmcrun_incomplete_2//Sottoriva/fina
CT_R4<-read.table("/media/nas/BitPhylogeny/methylation/backup/final_mcmcrun_incomplete_2//Sottoriva/fina
CT_R5<-read.table("/media/nas/BitPhylogeny/methylation/backup/final_mcmcrun_incomplete_2//Sottoriva/fina
CT_R6<-read.table("/media/nas/BitPhylogeny/methylation/backup/final_mcmcrun_incomplete_2//Sottoriva/fina

CT_L2<-read.table("/media/nas/BitPhylogeny/methylation/backup/final_mcmcrun_incomplete_2//Sottoriva/fina
CT_L3<-read.table("/media/nas/BitPhylogeny/methylation/backup/final_mcmcrun_incomplete_2//Sottoriva/fina
CT_L7<-read.table("/media/nas/BitPhylogeny/methylation/backup/final_mcmcrun_incomplete_2//Sottoriva/fina
CT_L8<-read.table("/media/nas/BitPhylogeny/methylation/backup/final_mcmcrun_incomplete_2//Sottoriva/fina

feature<-5
band<-0.2
g<-density(CT_R1[,feature],bw=band)

plot(g, col=rgb(0,0.7,0.7,1/2), main="CT max depth", xlim=c(min(CT_R1[,feature],CT_R4[,feature],CT_R5[,
        ylim=c(0,2),lwd=2, xaxt='n')
axis(1, at=c(2,3,4,5), labels=c(2,3,4,5))
lines(density(CT_R4[,feature],bw=band), col=rgb(0,0.8,0.8,1/2),lwd=2)
lines(density(CT_R5[,feature],bw=band), col=rgb(0,0.9,0.9,1/2),lwd=2)
lines(density(CT_R6[,feature],bw=band), col=rgb(0,1,1,1/2),lwd=2)

lines(density(CT_L2[,feature],bw=band), col=rgb(0.7,0,0.7,1/2),lwd=2)
```

```r
lines(density(CT_L3[,feature],bw=band), col=rgb(0.8,0,0.8,1/2),lwd=2)
lines(density(CT_L7[,feature],bw=band), col=rgb(0.9,0,0.9,1/2),lwd=2)
lines(density(CT_L8[,feature],bw=band), col=rgb(1,0,1,1/2),lwd=2)
```

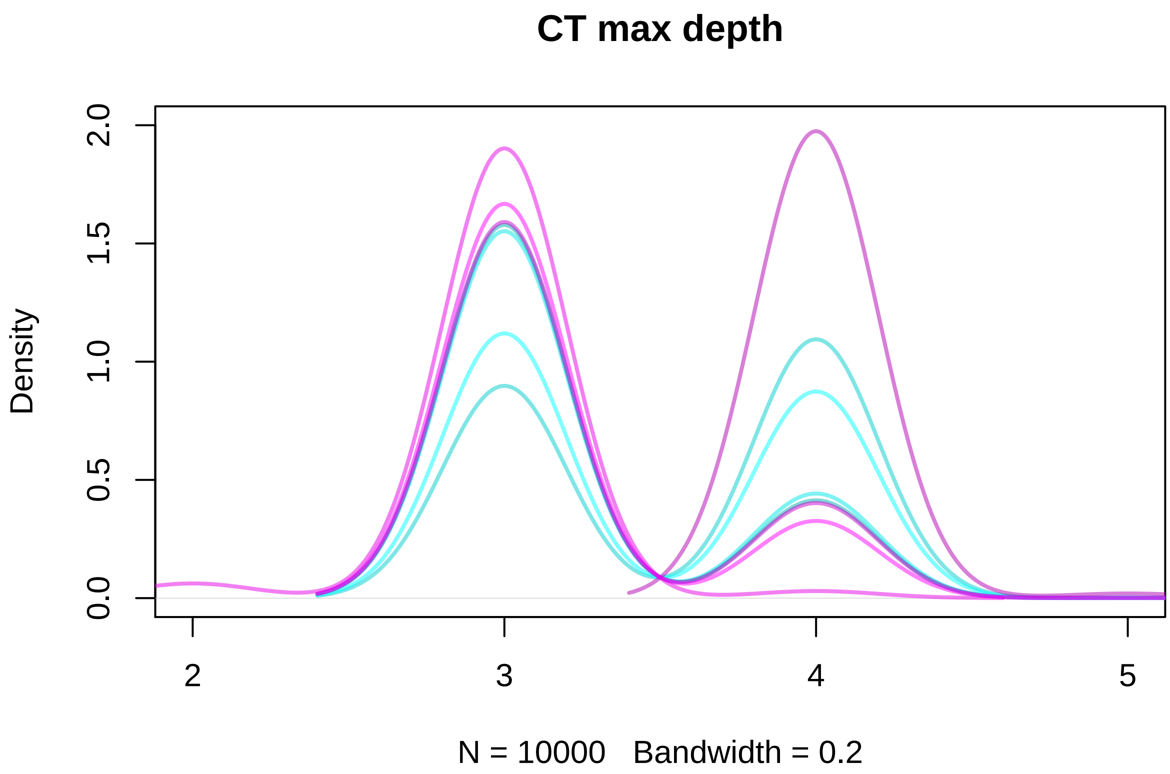


```r
wilcox.test(c(var(CT_R1[,feature]), var(CT_R4[,feature]), var(CT_R5[,feature]), var(CT_R6[,feature])),c
    alternative="greater")
```

```
##
##  Wilcoxon rank sum test
##
## data:  c(var(CT_R1[, feature]), var(CT_R4[, feature]), var(CT_R5[, feature]),  and c(var(CT_L2[, fea
## W = 16, p-value = 0.01429
## alternative hypothesis: true location shift is greater than 0
```

```r
feature<-23

massesR<-matrix(c(mean(CT_R1[,feature]),mean(CT_R1[,feature+1]),mean(CT_R1[,feature+2]),mean(CT_R1[,fea
        mean(CT_R5[,feature]),mean(CT_R5[,feature+1]),mean(CT_R5[,feature+2]),mean(CT_R5[,feature+3]),me
massesL<-matrix(c(mean(CT_L2[,feature]),mean(CT_L2[,feature+1]),mean(CT_L2[,feature+2]),mean(CT_L2[,fea
                mean(CT_L7[,feature]),mean(CT_L7[,feature+1]),mean(CT_L7[,feature+2]),mean(CT_L7[,featu
masses<-cbind(massesL,massesR)
masses<-apply(masses,2,rev)
par(mar=c(5.1,4.1,4.1,6.1))
barplot(masses,col=rev(c("red","blue","green","pink","orange")),names.arg=c("CT_L2", "CT_L3", "CT_L7",
        args.legend = list(x=11.5, y=1), bty = "n")
```

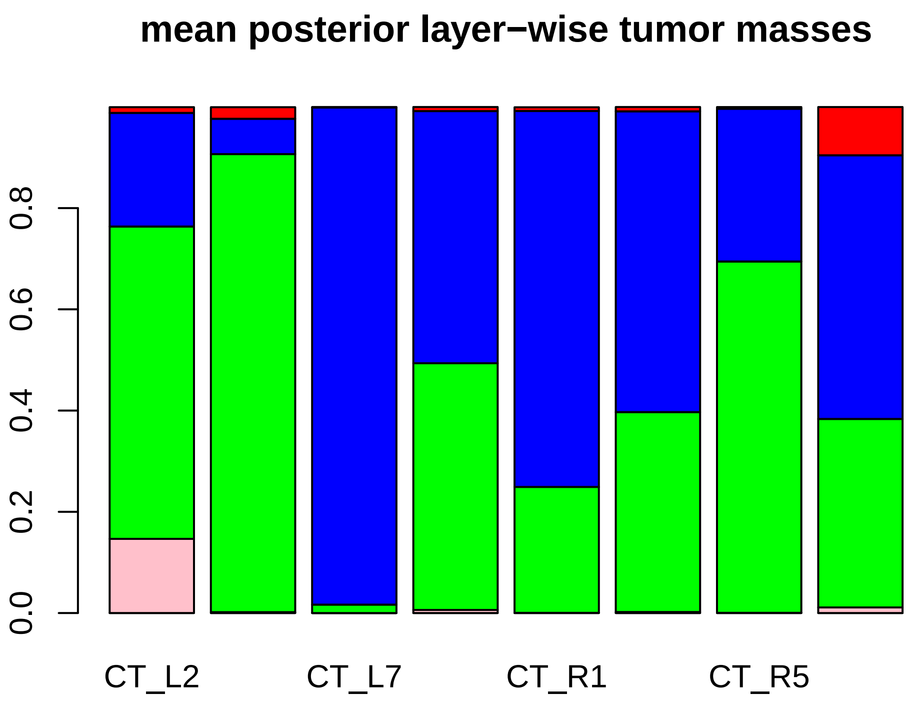


```
x<-0
massesR<-massesR+0.00000001
massesL<-massesL+0.00000001
rightDiff<-rep(0,6)
leftDiff<-rep(0,6)
for (j in 1:3 ) {
    for (i in (j+1):4) {
        x<-x+1
            rightDiff[x]<-(sum(log(massesR[,j]/massesR[,i])*massesR[,j])+sum(log(massesR[,i]/massesR[,j
            leftDiff[x]<-(sum(log(massesL[,j]/massesL[,i])*massesL[,j])+sum(log(massesL[,i]/massesL[,j]
        }
}
wilcox.test(rightDiff,leftDiff,alternative='less')
```

```
##
##  Wilcoxon rank sum test
##
## data:  rightDiff and leftDiff
## W = 1, p-value = 0.002165
## alternative hypothesis: true location shift is less than 0
```

6 Reproducing Figure 6

```
CX_R1<-read.table("/media/nas/BitPhylogeny/methylation/backup/final_mcmcrun_incomplete_2//Sottoriva/fina
CX_R2<-read.table("/media/nas/BitPhylogeny/methylation/backup/final_mcmcrun_incomplete_2//Sottoriva/fina
CX_R6<-read.table("/media/nas/BitPhylogeny/methylation/backup/final_mcmcrun_incomplete_2//Sottoriva/fina

CX_L3<-read.table("/media/nas/BitPhylogeny/methylation/backup/final_mcmcrun_incomplete_2/Sottoriva/final
```

```r
CX_L4<-read.table("/media/nas/BitPhylogeny/methylation/backup/final_mcmcrun_incomplete_2/Sottoriva/final
CX_L5<-read.table("/media/nas/BitPhylogeny/methylation/backup/final_mcmcrun_incomplete_2/Sottoriva/final


# #par(mfrow=c(1,2))
feature<-2
band<-0.7
g<-density(CX_R1[,feature],bw=band)

plot(g, col=rgb(0,0.7,0.7,1/2), lwd=2, main="CX node numbers", xlim=c(min(CX_R1[,feature],CX_R2[,feature
lines(density(CX_R2[,feature],bw=band), col=rgb(0,0.8,0.8,1/2),lwd=2)
lines(density(CX_R6[,feature],bw=band), col=rgb(0,0.9,0.9,1/2),lwd=2)

lines(density(CX_L3[,feature],bw=band), col=rgb(0.7,0,0.7,1/2),lwd=2)
lines(density(CX_L4[,feature],bw=band), col=rgb(0.8,0,0.8,1/2),lwd=2)
lines(density(CX_L5[,feature],bw=band), col=rgb(0.9,0,0.9,1/2),lwd=2)
```

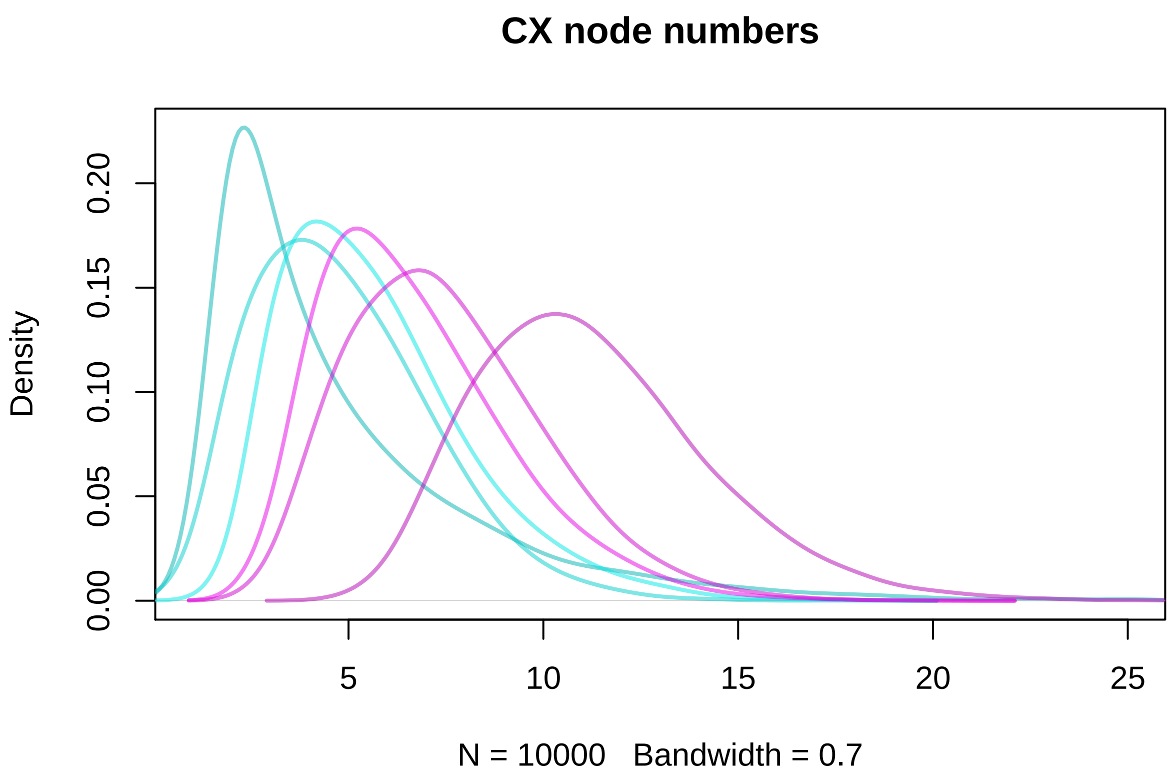


```r
#
#
 t.test(c(mean(CX_R1[,feature]),mean(CX_R2[,feature]),mean(CX_R6[,feature])),c(mean(CX_L3[,feature]),mea
```

```
##
##  Welch Two Sample t-test
##
## data:  c(mean(CX_R1[, feature]), mean(CX_R2[, feature]), mean(CX_R6[,  and c(mean(CX_L3[, feature]),
## t = -2.347, df = 2.173, p-value = 0.1334
## alternative hypothesis: true difference in means is not equal to 0
## 95 percent confidence interval:
```

```
##  -9.070  2.352
## sample estimates:
## mean of x mean of y
##     5.124     8.483
```

```
#
feature<-5
band<-0.2
g<-density(CX_R1[,feature],bw=band)

plot(g, col=rgb(0,0.7,0.7,1/2), main="CX max depth", xlim=c(min(CX_R1[,feature],CX_R2[,feature],CX_L3[,
        ylim=c(0,2),lwd=2, xaxt='n')
axis(1, at=c(1,2,3,4,5), labels=c(1,2,3,4,5))
lines(density(CX_R2[,feature],bw=band), col=rgb(0,0.8,0.8,1/2),lwd=2)
lines(density(CX_R6[,feature],bw=band), col=rgb(0,0.9,0.9,1/2),lwd=2)

lines(density(CX_L3[,feature],bw=band), col=rgb(0.7,0,0.7,1/2),lwd=2)
lines(density(CX_L4[,feature],bw=band), col=rgb(0.8,0,0.8,1/2),lwd=2)
lines(density(CX_L5[,feature],bw=band), col=rgb(0.9,0,0.9,1/2),lwd=2)
```

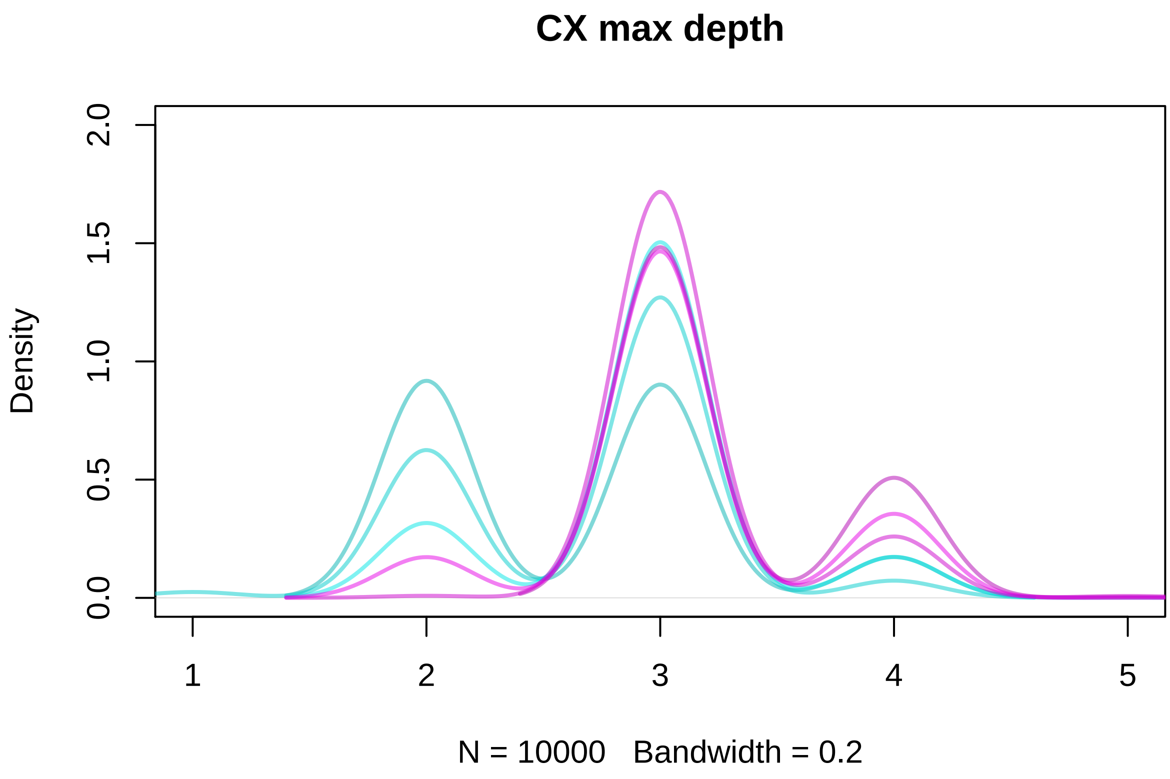


```
#
t.test(c(mean(CX_R1[,feature]),mean(CX_R2[,feature]),mean(CX_R6[,feature])),c(mean(CX_L3[,feature]),mea
```

```
##
##  Welch Two Sample t-test
##
## data:  c(mean(CX_R1[, feature]), mean(CX_R2[, feature]), mean(CX_R6[,  and c(mean(CX_L3[, feature]),
## t = -3.959, df = 3.099, p-value = 0.02711
```

```
## alternative hypothesis: true difference in means is not equal to 0
## 95 percent confidence interval:
##  -0.73421 -0.08632
## sample estimates:
## mean of x mean of y
##     2.751     3.161
```

```
#
#
#


feature1<-2
feature2<-5
plot(mean(CX_R1[,feature1]),mean(CX_R1[,feature2]),xlim=c(4,12),ylim=c(2.5,3.3),col=rgb(0,0.7,0.7,1/2),
points(mean(CX_R2[,feature1]),mean(CX_R2[,feature2]),col=rgb(0,0.7,0.7,1/2),lwd=4,cex=3)
points(mean(CX_R6[,feature1]),mean(CX_R6[,feature2]),col=rgb(0,0.8,0.8,1/2),lwd=4,cex=3)

points(mean(CX_L3[,feature1]),mean(CX_L3[,feature2]),col=rgb(0.7,0,0.7,1/2),lwd=4,pch=2,cex=3)
points(mean(CX_L4[,feature1]),mean(CX_L4[,feature2]),col=rgb(0.8,0,0.8,1/2),lwd=4,pch=2,cex=3)
points(mean(CX_L5[,feature1]),mean(CX_L5[,feature2]),col=rgb(0.9,0,0.9,1/2),lwd=4,pch=2,cex=3)
```

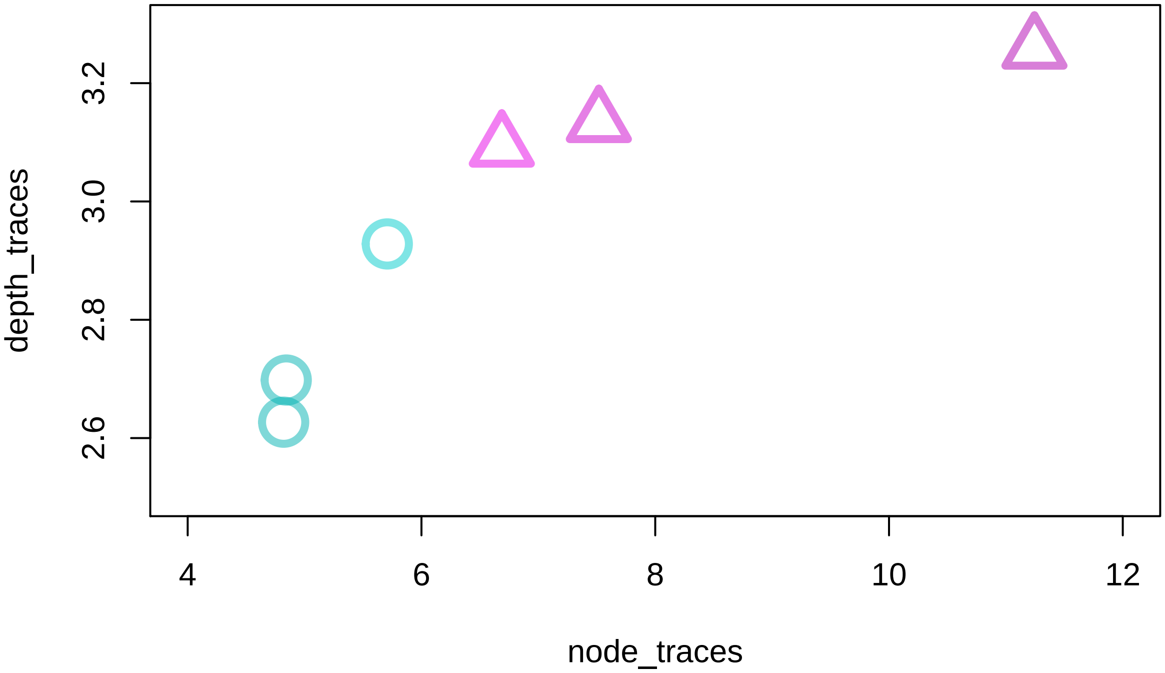

7 Reproducing Figure 7 and S3

```
library(e1071)
load("~/Dropbox/bitphylogeny_pkg/single_cell/params_summary.Rdata")

snv <- read.csv("~/Dropbox/bitphylogeny_pkg/single_cell/snv.csv", as.is = T)
```

```
cellNames <- colnames(snv)[-1]

geneList <- read.table("~/Dropbox/bitphylogeny_pkg/single_cell/Outfile_short.csv", header = T, as.is =T)

bed <- read.csv('~/Dropbox/bitphylogeny_pkg/single_cell/hou_data_bed.csv', as.is = T)

indexSESN2 <- geneList[which(geneList[,"GeneSym"] == "SESN2"),
                       c("Chrom", "Start", "GeneSym")]

indexNTRK1 <- geneList[which(geneList[,"GeneSym"] == "NTRK1"),
                       c("Chrom", "Start", "GeneSym")]

indexST13 <- geneList[which(geneList[,"GeneSym"] == "ST13"),
                      c("Chrom", "Start", "GeneSym")]

indexABCB5 <- geneList[which(geneList[,"GeneSym"] == "ABCB5"),
                       c("Chrom", "Start", "GeneSym")]

indexFRG1 <- geneList[which(geneList[,"GeneSym"] == "FRG1"),
                      c("Chrom", "Start", "GeneSym")]

indexASNS <- geneList[which(geneList[,"GeneSym"] == "ASNS"),
                      c("Chrom", "Start", "GeneSym")]

indexTOP1MT <- geneList[which(geneList[,"GeneSym"] == "TOP1MT"),
                        c("Chrom", "Start", "GeneSym")]

indexDNAJC17 <- geneList[which(geneList[,"GeneSym"] == "DNAJC17"),
                         c("Chrom", "Start", "GeneSym")]

indexDF <- rbind(indexSESN2, indexNTRK1, indexST13, indexABCB5, indexFRG1,
                 indexASNS, indexTOP1MT, indexDNAJC17)

rownames(indexDF) <- NULL

ll <- mapLabels[1,]
rownames(ll) <- NULL
colnames(ll) <- NULL

ll <- as.character(ll)
ll <- revalue(ll, c("0"="a", "2"="c", "10"="f", "11"="g", "5"="i",
              "7"="d", "4"="h", "8"="e", "1"="b"))


FindGeneIndex <- function(x, y){
  ind <- c()
  for (i in 1:dim(y)[1]) {
    tmp <- sapply(1:dim(x)[1], function(j) sum(x[j,] == y[i,]) == 2)
    ind <- c(ind, which(tmp == T))
  }
  return(ind)
}
```

```r
GetGeneDataFrame <- function (bed, index, snv, ll, meanParams, sdParams) {
  x <- data.frame()
  for (i in 1 : dim(index)[1]) {

    ind <- FindGeneIndex(bed[, c(1,2)], index[i, c(1,2)])

    meanValues <- meanParams[ind, ]
    sdValues <- sdParams[ind, ]
    snvData <- t(snv[ind, -1])
    rownames(snvData) <- NULL
    colnames(snvData) <- NULL
    snvData[is.na(snvData)] <- 2

    x <- rbind(x,
               data.frame(mean= meanValues,
                          sd = sdValues,
                          cell = 1:60,
                          snv = as.factor(snvData),
                          clone = as.factor(t(ll)),
                          gene = paste(index[i, 3],
                                       index[i, 1],
                                       index[i, 2],
                                       sep = ",") ) )
  }
  return(x)
}

dfall <- GetGeneDataFrame(bed, indexDF, snv, ll, meanParams, sdParams)

print(ggplot(dfall, aes(x=cell, y=sigmoid(mean), color = clone)) +
  geom_errorbar(aes(ymin= sigmoid(mean-sd), ymax=sigmoid(mean+sd)), width=1) +
  geom_point(size=2.5) + ylab("probability of mutation") +
  facet_wrap(~gene, ncol=2) + theme_bw() +
  theme(axis.text.x  = element_text(vjust=0.5, size = 16),
        axis.title.x = element_text(face="bold", size = 20),
        axis.text.y = element_text(size = 16),
        axis.title.y = element_text(face="bold", size = 20),
        legend.title = element_text(face="bold",size = 18),
        legend.text = element_text(face="bold",size = 18),
        strip.text.x = element_text(face="bold",size = 18))
  )
```

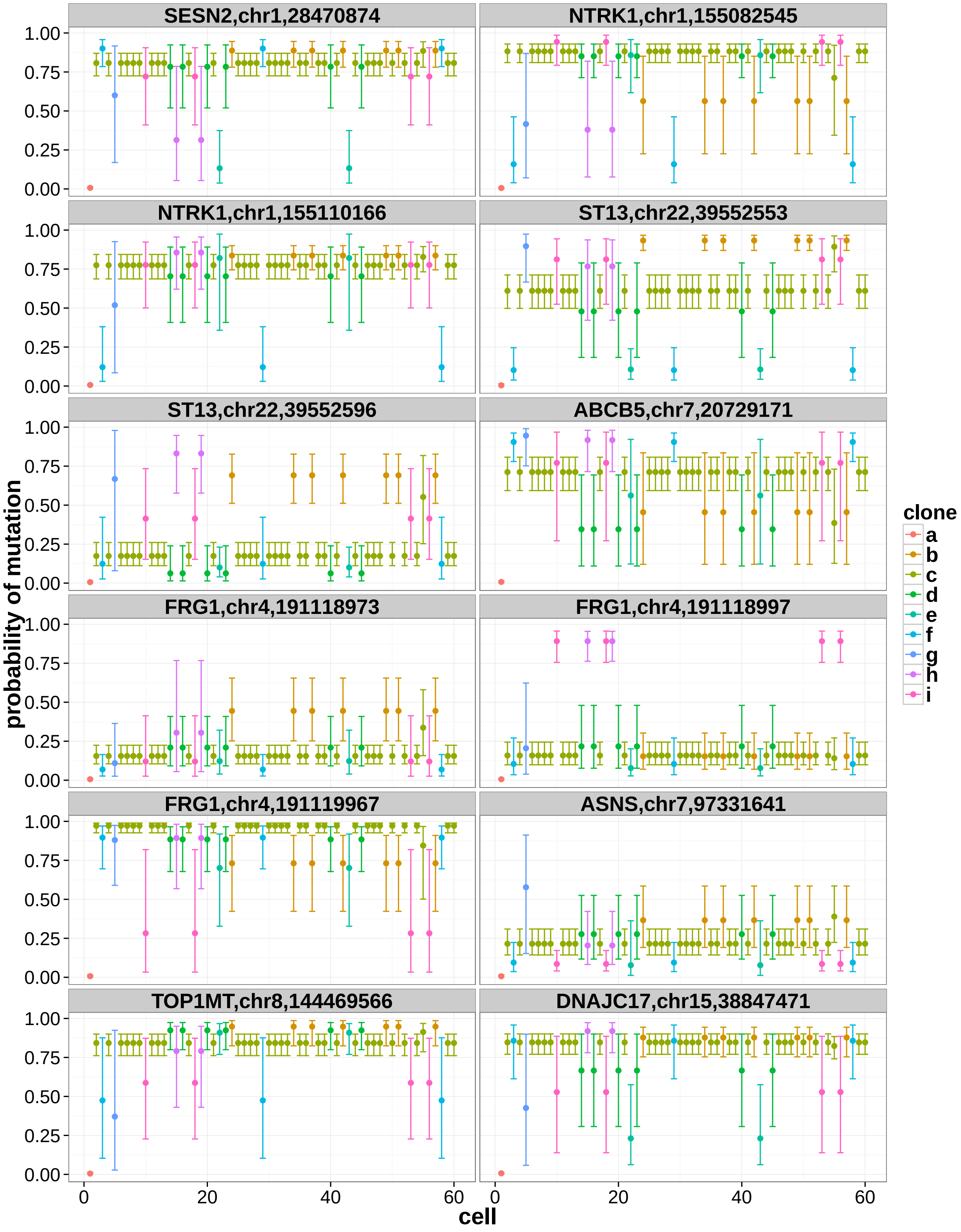
SESN2,chr1,28470874
NTRK1,chr1,155082545
NTRK1,chr1,155110166
ST13,chr22,39552553
ST13,chr22,39552596
ABCB5,chr7,20729171
FRG1,chr4,191118973
FRG1,chr4,191118997
FRG1,chr4,191119967
ASNS,chr7,97331641
TOP1MT,chr8,144469566
DNAJC17,chr15,38847471
probability of mutation
cell
clone
a
b
c
d
e
f
g
h
i
0.00
0.25
0.50
0.75
1.00
0
20
40
60

8 Reproducing Figure S1

```
library(bitphylogenyR)
library(corrplot)
library(psych)
```

```
##
## Attaching package: 'psych'
##
## The following object is masked from 'package:ggplot2':
##
##     %+%
```

```
SetLociCorr <- function(mode = "IRX2P"){
  files <- dir(system.file('extdata/sottoriva/', package = "bitphylogenyR"),
               full.names = T, pattern = mode)
  if (mode == "IRX2P") {
    n = 8
  } else if (mode == "SLC5A7P") {
    n = 6
  } else {
    n = 16
  }

  corrmat <- array(0, dim = c(n, n))
  nonnacount <- array(0, dim = c(n, n))

  for (i in 1:length(files)) {
    x <- read.csv(files[i])
    t1 <- corr.test(unique(x), method = "kendall")
    t2 <- t1$p <= 0.05
    t2[which(is.na(t2))] <- FALSE
    corrmat <- corrmat + t2
    nonnacount <- nonnacount + !is.na(t1$p <= 0.05)
  }

  corrmat[lower.tri(corrmat)] <- 0
  corrmat <- corrmat + t(corrmat)
  diag(corrmat) = diag(corrmat) / 2
  M <- corrmat / nonnacount
  return( M )
}

M1 <- SetLociCorr(mode = "IRX2P")
M2 <- SetLociCorr(mode = "SLC5A7P")
M3 <- SetLociCorr(mode = "ZNF454")

mat <- matrix(c(1,3,3,2,3,3), 3)

layout(mat)
corrplot(M1, method = "pie", is.corr=F,
         cl.lim = c(0,1), type = "upper", tl.pos = "d", cl.pos = "n")
```

```
title("IRX2P", line = -11, cex.main = 2)
corrplot(M2, method = "pie", is.corr=F,
         cl.lim = c(0,1), type = "upper", tl.pos = "d", cl.pos = "n")
title("SLC5A7P",  line = -11, cex.main = 2)
corrplot(M3, method = "pie", is.corr=F,
         cl.lim = c(0,1), type = "upper", tl.pos = "d")
title("ZNF454",  line = -21, cex.main = 2)
```

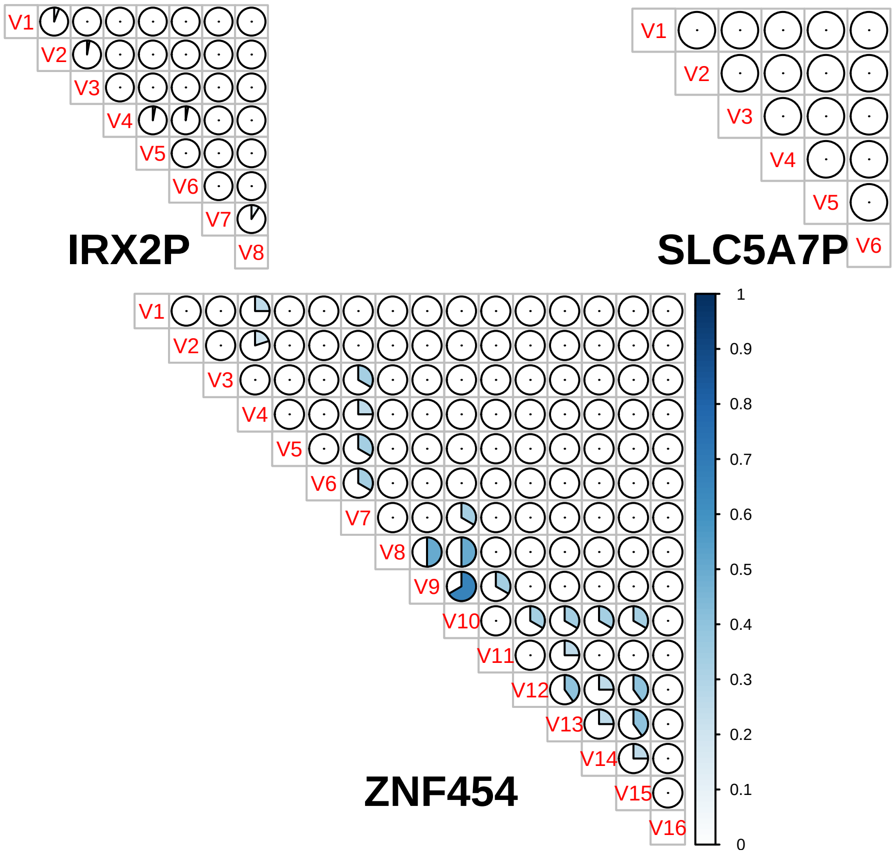

Supplement: Additional file 2 — R markdown file. A PDF file with BitPhylogeny package details and figure reproduction. [file 13059_2015_592_MOESM2_ESM.pdf]
